# Stereoselective effects of nicotine enantiomers on the gut-brain axis and neuroinflammation in a mouse model of Parkinson’s disease

**DOI:** 10.3389/fnagi.2026.1823372

**Published:** 2026-05-13

**Authors:** Ruixia Liu, Yunu Ma, Yingyan Li, Xia Gao, Shu Wang, Lutao Xu, Guanglin Liu, Huan Chen, Min Ji, Hongwei Hou

**Affiliations:** 1 Hefei Institutes of Physical Science, Chinese Academy of Sciences, Hefei, China; 2 University of Science and Technology of China, Hefei, China; 3 Beijing Life Science Academy, Beijing, China; 4 Key Laboratory of Tobacco Biological Effects, China National Tobacco Quality Supervision & Test Center, Zhengzhou, China

**Keywords:** *Akkermansia*, gut microbiota, gut-brain axis, MPTP mouse model, neuroinflammation, nicotine enantiomers, Parkinson’s disease, stereoselectivity

## Abstract

**Introduction:**

Parkinson’s disease (PD) is characterized by progressive dopaminergic neurodegeneration, neuroinflammation, and emerging evidence of gut microbiota dysbiosis. Although nicotine has been implicated in neuroprotection, whether its enantiomers exert stereoselective effects on the gut-brain axis remains unknown.

**Methods:**

We systematically compared the effects of S-nicotine and R-nicotine in an MPTP-induced mouse model of PD. Motor function, nigrostriatal dopaminergic neuronal loss, neuroinflammatory responses, intestinal barrier integrity, and gut microbiota composition were assessed.

**Results:**

Both enantiomers ameliorated motor deficits, attenuated nigrostriatal dopaminergic neuronal loss, and suppressed neuroinflammatory responses, yet exhibited markedly different efficacy profiles. S-nicotine produced robust neuroprotection at a lower effective dose (1 mg/kg), associated with improved motor performance, reduced central and peripheral inflammation, preservation of intestinal barrier integrity, and coordinated remodeling of the gut microbiota characterized by enrichment of Akkermansiaceae (notably *Akkermansia*). In contrast, R-nicotine required a higher dose (3 mg/kg) to achieve comparable neuroprotection and induced weaker and more heterogeneous microbiota alterations. Correlation analyses further revealed that S-nicotine-associated enrichment of *Akkermansia* was closely linked to improvements in motor function, reduced inflammatory status, and enhanced intestinal integrity.

**Discussion:**

These findings demonstrate pronounced stereoselective and dose-dependent effects of nicotine enantiomers on neuroimmune modulation and identify gut microbiota remodeling as a functionally relevant correlate of nicotine-mediated neuroprotection in PD. Our results highlight the therapeutic potential of targeting the microbiota-gut-brain axis through chiral interventions and support S-nicotine as a promising candidate for neuroprotective strategies in aging-related neurodegenerative disorders.

## 1 Introduction

Parkinson’s disease (PD) is the world’s second most common neurodegenerative disorder, characterized by the gradual progression of motor impairments such as bradykinesia, rigidity, and instability in posture (Tolosa et al., 2021). The pathological hallmark of PD includes the loss of dopaminergic neurons in the substantia nigra pars compacta (SNpc) and the aggregation of misfolded α-synuclein (Morris et al., 2024). Present therapeutic approaches are primarily centered on dopamine, offering symptom relief without preventing disease progression (McFarthing et al., 2021; Rai et al., 2021). Growing evidence indicates that Parkinson’s disease extends beyond the central nervous system, involving systemic interactions between neural, immune, and gastrointestinal systems, emphasizing the role of non-neuronal processes in disease progression (Houser and Tansey, 2017; Morris et al., 2024).

Recently, the bidirectional relationship between the microbiota, gut, and brain has become an important aspect of understanding PD pathophysiology (Houser and Tansey, 2017). Clinical and experimental research has consistently found gastrointestinal issues, compromised intestinal barrier integrity, and gut microbiota imbalance in PD patients, often occurring before motor symptoms appear (Scheperjans et al., 2014; Keshavarzian et al., 2015; Sampson et al., 2016; Lai et al., 2018). Disruption of the intestinal epithelium may facilitate the translocation of microbial products and pro-inflammatory mediators, which has been associated with peripheral immune activation and neuroinflammatory responses (Sampson et al., 2016; Houser and Tansey, 2017). These observations suggest that alterations in gut homeostasis are closely linked to immune and neural processes relevant to PD.

Nicotine, a naturally occurring alkaloid present in Solanaceae plants such as tomatoes and goji berries (Siegmund et al., 1999) and the principal bioactive component of tobacco, has long attracted attention due to epidemiological observations reporting an inverse association between smoking and PD risk (Hernán et al., 2002; Ritz et al., 2007; Mappin-Kasirer et al., 2020). Experimental studies have further demonstrated that nicotine exerts neuroprotective, anti-inflammatory, and neuromodulatory effects, primarily through activation of nicotinic acetylcholine receptors (nAChRs) (Wang et al., 2002; Dajas-Bailador and Wonnacott, 2004; Quik et al., 2006; Quik et al., 2013). Beyond its direct neuronal actions, nicotine has been reported to suppress pro-inflammatory cytokine production in both peripheral immune cells and central glial populations (Liu et al., 2012; Navarro et al., 2021), implicating its potential to modulate both systemic and neuroinflammatory processes relevant to PD.

Importantly, nicotine is a chiral molecule consisting of two enantiomers, L-(−)-nicotine (S-nicotine) and D-(+)-nicotine (R-nicotine), which exhibit distinct pharmacological properties (Yang et al., 2019). Naturally occurring S-nicotine displays substantially higher affinity and efficacy toward nAChRs than its R-enantiomer, resulting in pronounced stereoselectivity in receptor binding, signal transduction, and biological activity (Pogocki et al., 2007). While S-nicotine has been extensively studied in the context of neuroprotection and inflammation (Quik et al., 2006; Quik et al., 2013), the biological actions of R-nicotine remain relatively underexplored, particularly with respect to dose-dependent effects and its potential influence on peripheral inflammation and gut-associated processes. Whether the two nicotine enantiomers exert distinct or overlapping effects on PD-associated neuroinflammation, intestinal pathology, and gut microbiota composition remains to be systematically evaluated.

In this study, we systematically investigated the stereoselective effects of S-nicotine and R-nicotine across multiple dose gradients in a 1-methyl-4-phenyl-1,2,3,6-tetrahydropyridine (MPTP)-induced mouse model of PD. By integrating behavioral assessments, dopaminergic neuronal analysis, multi-compartment inflammatory profiling, evaluation of intestinal barrier integrity, and 16S rRNA-based gut microbiota characterization, we aimed to delineate the dose-dependent and enantiomer-specific effects on neurodegeneration, inflammation, and microbiota-host interactions. Our findings provide new insights into the stereoselective actions of nicotine and support the gut-brain axis as a functionally relevant component of nicotine-associated neuroimmune modulation in PD.

## 2 Materials and methods

### 2.1 Animals

C57BL/6 N mice, male, aged 8–10 weeks, were obtained from Beijing Vital River Laboratory Animal Technology Co., Ltd. (Beijing, China). Mice were housed under specific pathogen-free (SPF) conditions (24 ± 2 °C, 55 ± 10% humidity) with a 12 h light/dark cycle and free access to chow and water. All animals were acclimatized for 1 week prior to experimental manipulation. All experimental procedures were approved by the Laboratory Animal Management and Ethics Committee of the China National Tobacco Quality Supervision and Test Center (Approval No. CTQTC-SYXK-2025001) and conformed to the National Institutes of Health Guide for the Care and Use of Laboratory Animals.

### 2.2 Reagents

MPTP hydrochloride (CAS: 23007–85-4, Item NO.: ST1020-100 mg, ≥98%, reagent grade) was purchased from Beyotime Biotechnology (Shanghai, China). Nicotine (purity > 99%) was provided by the Key Laboratory of Tobacco Biological Effects (Zhengzhou, China). All other chemicals and reagents were of analytical grade.

### 2.3 Experimental design

After 1 week of acclimatization, mice were randomly assigned to experimental groups using a random number table. The sample size for each group was determined based on pilot experiments and previous studies, with *n* = 5–11 mice per group for behavioral tests, *n* = 3–11 mice per group for biochemical analyses, and *n* = 6–7 mice per group for microbiota sequencing.

Mice were divided into the following groups: vehicle (Con), MPTP, S-nicotine (S-NIC), and R-nicotine (R-NIC). For MPTP groups, MPTP (25 mg/kg, i.p.) was administered once daily for 7 days to induce PD-like pathology, following a previously published subacute protocol (Meredith and Rademacher, 2011; Li et al., 2025b). Control mice received saline. Nicotine enantiomers were administered by subcutaneous (s.c.) injection daily at indicated doses: S-NIC at 0.25, 0.5, or 1 mg/kg; R-NIC at 0.25, 0.5, 1, or 3 mg/kg. Nicotine treatment began 7 days before the first MPTP injection and continued throughout the MPTP induction period. Vehicle and MPTP groups received equivalent volumes of saline s.c. as controls. All nicotine-treated groups were administered in combination with MPTP unless otherwise specified. All behavioral tests and sample collections were performed by experimenters blinded to group allocation.

### 2.4 Behavioral tests

All behavioral procedures were conducted between 9:00 a.m. and 3:00 p.m. in a quiet, dimly lit room. Mice were brought to the testing room 30 min before each test for habituation. Apparatuses were cleaned with 75% ethanol between trials to eliminate olfactory cues.

- Rotarod Test: Motor coordination and balance were evaluated using an accelerating rotarod apparatus (YLS-4C, Shanghai Jinan, China). Mice were placed on the rotarod, which accelerated from 4 to 30 rpm over 120 s. Each mouse underwent three training trials on the day before testing, followed by three test trials with 1 h inter-trial intervals. The latency to fall was recorded automatically, and the mean of the three test trials was used for statistical analysis.
- Open Field Test: Locomotor activity and anxiety-like behavior were assessed in an open field arena (50 cm × 50 cm × 40 cm) equipped with an overhead camera. At the start of the trial, each mouse was placed against the wall of the arena and allowed to explore freely for 10 min. Total distance traveled, distance traveled in the central zone (25 × 25 cm), and time spent in the central zone were recorded using a video tracking system (Xinruan Information Technology, Shanghai, China). The arena was illuminated at approximately 60 lux.
- Elevated Plus-Maze Test: Anxiety-related behavior was further examined using an elevated plus-maze consisting of two open arms (30 × 5 cm) and two enclosed arms (30 × 5 × 15 cm) extending from a central platform (5 × 5 cm), elevated 50 cm above the floor. Mice were placed in the center facing an open arm and allowed to explore for 5 min. The time spent in the open arms was recorded using the video tracking system. Illumination was maintained at approximately 40 lux in the open arms.
- Gait Analysis: Gait characteristics were evaluated using an automated gait analysis system (CatWalk XT, Noldus Information Technology, The Netherlands). Each mouse was trained once daily for 3 consecutive days prior to formal testing, with each session lasting approximately 3 min and gradually increasing in walking speed. During testing, each mouse was encouraged to traverse the glass walkway from the start to the dark goal box, and only uninterrupted runs with stable speed were included. System calibration and illumination adjustments were performed prior to testing. Gait parameters, including stride length, stance time, and swing time, were analyzed automatically using the CatWalk XT software.

### 2.5 Immunofluorescent staining

Mice were deeply anesthetized with 2,2,2-tribromoethanol (0.2 mL/kg, i.p., JT0791, Beijing Jitian, China) and perfused transcardially with saline followed by 4% paraformaldehyde (PFA) in 0.1 mol/L phosphate-buffered saline (PBS). Brain and distal colon tissues were fixed in 4% PFA for 6–8 h at 4 °C, dehydrated sequentially in 15 and 30% sucrose solutions at 4 °C until sinking. Tissues were embedded in optimal cutting temperature (OCT) compound (Sakura Finetek, Torrance, CA, USA) and coronally sectioned at 20 μm thickness using a cryostat (Leica CM1950, Leica Biosystems, Nussloch, Germany). Sections were washed with PBS, blocked with PBS containing 0.5% Triton X-100 and 5% BSA for 30 min at room temperature, and incubated overnight at 4 °C with rabbit anti-tyrosine hydroxylase (TH, 1:200, AB152, Merck Millipore, MA, USA). After washing, sections were incubated with Alexa Fluor® 488-conjugated secondary antibody (Goat anti-rabbit IgG, 1:200, ab205718, Abcam, Cambridge, UK) for 1 h at room temperature in the dark. Sections were mounted with DAPI-containing mounting medium (Vector Laboratories, Burlingame, CA, USA). Images were captured using a Pannoramic MID digital slide scanner (3DHISTECH Ltd., Budapest, Hungary). TH-positive neurons in the substantia nigra pars compacta (SNpc) were quantified bilaterally in three coronal sections per mouse using ImageJ software (National Institutes of Health, Bethesda, MD, USA) by an experimenter blinded to group allocation.

### 2.6 Hematoxylin and eosin staining

Colon tissues were fixed in 4% PFA, paraffin-embedded, and cut into 5 μm slices. Histological staining was performed using a hematoxylin and eosin (H&E) staining kit (Servicebio, G1076, Wuhan, China). Stained sections were scanned using a whole slide imaging (WSI) scanner (Servicebio, LG-S80) for histopathological analysis.

### 2.7 Sample collection and preparation

Mice were deeply anesthetized as described above. Blood was collected from retro-orbital sinus and allowed to clot at room temperature for 60 min. Serum was separated by centrifugation at 3000 rpm for 10 min at 4 °C, aliquoted, and stored at −80 °C until analysis. After blood collection, mice were euthanized by decapitation. Brains were rapidly removed and placed on an ice-cold plate. The SN, striatum, and hippocampus were dissected bilaterally according to a mouse brain atlas. A segment of distal colon (approximately 1 cm in length) was also dissected and flushed with ice-cold PBS to remove luminal contents. All tissue samples were immediately snap-frozen in liquid nitrogen and stored at −80 °C until further processing.

### 2.8 Inflammatory cytokine analysis

The concentrations of inflammatory cytokines, including tumor necrosis factor-α (TNF-α), interleukin-1β (IL-1β), and interleukin-6 (IL-6), in serum and colon were quantitatively measured using commercial ELISA kits (Jianglai Bio, China) according to the manufacturer’s instructions. The protein levels of TNF-α, IL-1β, and IL-6 in the SN and striatum were determined using the RayPlex™ Mouse Inflammation Array 1 (FAM-INF-1-96, RayBiotech, USA), following the manufacturer’s instructions.

### 2.9 Western blot analysis

Tissue samples from SN and hippocampus were homogenized in RIPA lysis buffer (P0013B, Beyotime, Shanghai, China) supplemented with protease and phosphatase inhibitor cocktails (P1045, Beyotime, Shanghai, China). Homogenates were incubated on ice for 30 min and centrifuged at 12,000 rpm for 5 min at 4 °C, and the supernatant was collected. Protein concentrations were determined using the BCA assay. The remaining supernatant was mixed with 5 × loading buffer, boiled for 10 min, and stored at −20 °C.

Equal amounts of protein (20 μg per lane) were separated by 10% SDS-PAGE and transferred onto polyvinylidene difluoride (PVDF) membranes (Millipore, Burlington, MA, USA). Membranes were blocked a Western Blocking Buffer (P0023B, Beyotime, China) for 2 h at room temperature, followed by overnight incubation at 4 °C with primary antibodies: rabbit anti-TH (1:5000, AB152, Merck Millipore, Burlington, MA, USA); rabbit anti-TNF-α (1:2000, 17,590-1-AP, Proteintech, Wuhan, China); rabbit anti-IL-1β (1:2000, 26,048-1-AP, Proteintech, Wuhan, China); rabbit anti-IL-6 (1:2000, 26,404-1-AP, Proteintech, Wuhan, China); rabbit anti-GFAP (1:5000, ab68428, Abcam, Cambridge, UK); rabbit anti-Iba1 (1:1000, ab178847, Abcam, Cambridge, UK); rabbit anti-GAPDH (1:1000, K200057M, Solarbio, Beijing, China); or rabbit anti-β-actin (1:1000, K112355P, Solarbio, Beijing, China). After washing with TBST (3 × 10 min), membranes were incubated with HRP-conjugated goat anti-rabbit IgG secondary antibody (1:2000, ab205718, Abcam, Cambridge, UK) for 2 h at room temperature. Bands were visualized using an ECL Luminescence Kit (Beyotime, P0018AS) and visualized with the Tanon-5200 Multi chemiluminescent imaging system (Tanon Science & Technology Co., Ltd., Shanghai, China). Densitometric analysis was performed using ImageJ software (National Institutes of Health, USA), and target protein levels were normalized to β-actin or GAPDH.

### 2.10 RT-qPCR analysis

Total RNA from the striatum was extracted using the SteadyPure Universal RNA Extraction Kit (Accurate Biology, Changsha, China) according to the manufacturer’s protocol. RNA concentration and purity were assessed using a NanoDrop 2000 spectrophotometer (Thermo Fisher Scientific, Waltham, MA, USA). cDNA was synthesized from 1 μg total RNA using the Evo M-MLV RT Premix Kit with gDNA removal. RT-qPCR was performed with SYBR® Green Pro Taq HS Premix (Accurate Biology, Changsha, China) on a LightCycler® 480 system (Roche, Basel, Germany). Relative mRNA levels were normalized to GAPDH using the 2^−ΔΔCt^ method and normalized to GAPDH as the internal control. Primer sequences are listed below: (1) *Tnf-α*-F: GATCGGTCCCCAAAGGGATG; −R: GCTCCTCCACTTGGTGGTTT; (2) *IL-1β*-F: TGCCACCTTTTGACAGTGATG; −R: TGATGTGCTGCTGCGAGATT; (3) *IL-6-F*: AGTCCGGAGAGGAGACTTCA; −R: GCCATTGCACAACTCTTTTCT; (4) *ZO-1-F*: GCTTTAGCGAACAGAAGGAGGC; −R: TTCATTTTTCCGAGACTTCACCA; (5) *Occludin*-F: TGAAAGTCCACCTCCTTTACAGA; −R: CCGGATAAAAAAGAGTACGCTGG.

### 2.11 *Ex vivo* nicotine pharmacokinetics

After intraperitoneal injection (1 mg/kg S-nicotine, 3 mg/kg R-nicotine, or saline), brain tissues (striatum, hippocampus, and SN), blood samples, and colon tissues were collected. Brain and colon tissues were immediately frozen in liquid nitrogen and stored at −80 °C. Blood samples were placed in 1.5 mL Eppendorf tubes containing 5 μL of heparin sodium solution (10 g/L), thoroughly mixed, and centrifuged at 4,000 r/min for 10 min. The separated plasma was stored at −40 °C until analysis.

Concentrations of S- and R-nicotine in brain tissue, plasma, and colon tissue were determined using an ultra-high performance liquid chromatography–tandem mass spectrometry (UPLC-MS/MS) method as previously described. The optimized chromatographic conditions were as follows: separation was achieved on a Chiralpak IG-3 chiral column (250 mm × 4.6 mm × 3 μm) coupled with a guard column (10 mm × 4.0 mm × 3 μm). The column temperature was maintained at 30 °C. The mobile phase consisted of 0.2% ammonium formate in methanol at a flow rate of 1.2 mL/min. The injection volume was 10 μL, and the run time was 15 min.

### 2.12 16S rRNA sequencing

Cecal contents were collected immediately after euthanasia, snap-frozen in liquid nitrogen, and stored at −80 °C until DNA extraction. Total microbial genomic DNA was extracted using the QIAamp Fast DNA Stool Mini Kit (Qiagen, Hilden, Germany) following the manufacturer’s instructions. DNA concentration and purity were assessed by NanoDrop 2000, and DNA integrity was verified by 1% agarose gel electrophoresis.

The V3-V4 region of the 16S rRNA gene was amplified, and sequenced on an Illumina platform. Raw reads were quality-filtered and processed with DADA2 for denoising, chimera removal, and ASV generation. Taxonomy was assigned using the Silva database. α diversity was assessed by ACE and Shannon indices, and β diversity by PCoA based on unweighted UniFrac distances. Taxonomic differences were analyzed using LEfSe and visualized with cladograms.

### 2.13 Statistical analysis

Statistical analyses were performed using GraphPad Prism 9.0 (GraphPad Software, San Diego, CA, USA). Data are presented as mean ± standard error of the mean (SEM). Normality of data distribution was assessed by Shapiro–Wilk test. For comparisons between any two groups on other endpoints (e.g., behavioral and biochemical), the unpaired Student’s *t*-test were performed for normally distributed data, and Mann–Whitney U test was used for non-normally distributed data. For comparisons involving multiple groups, one-way analysis of variance (ANOVA) was performed, followed by Dunnett’s *post hoc* test for comparisons against the MPTP group. For body weight changes over time, two-way repeated measures ANOVA was used, followed by Dunnett’s *post hoc* test. Correlation analyses between gut microbiota abundance and PD-related parameters were performed using Spearman’s rank correlation coefficient. *p* value < 0.05 was considered statistically significant. Sample sizes (n) are indicated in figure legends.

## 3 Results

### 3.1 MPTP administration induces motor deficits, dopaminergic neuronal loss, neuroinflammation, and gut pathology in mice

**Figure 1:**
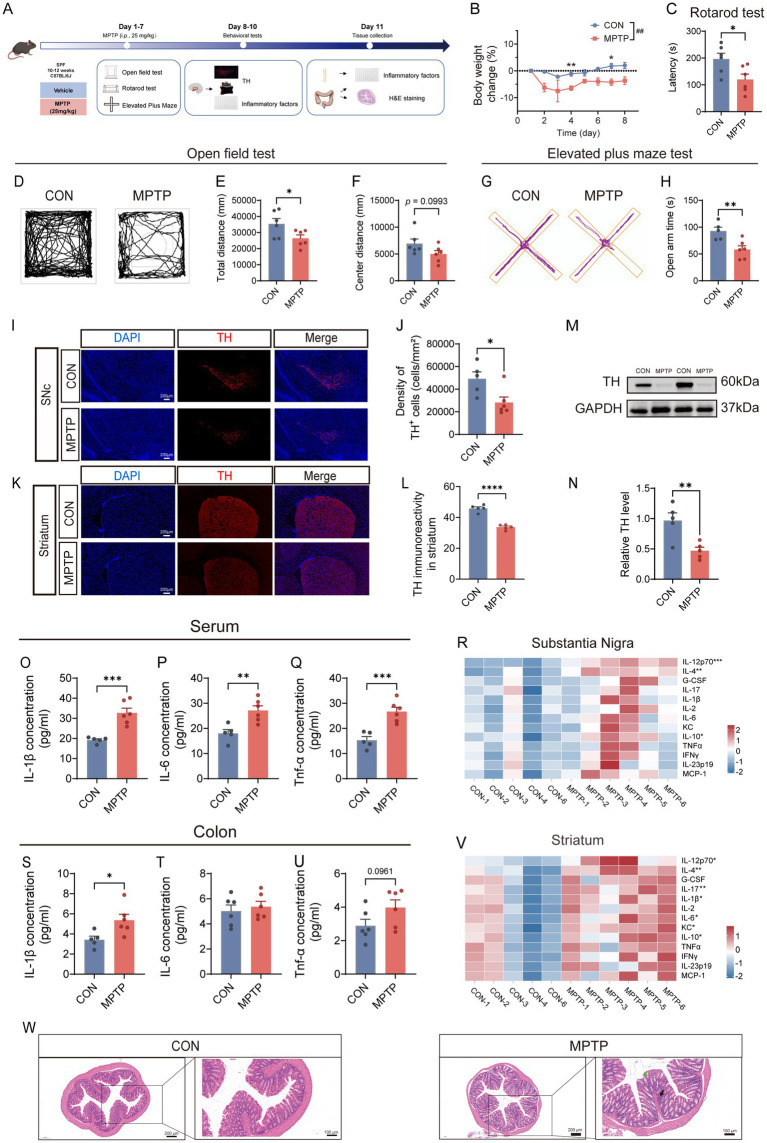
MPTP treatment induces motor deficits, dopaminergic neuronal loss, neuroinflammation, and colonic pathology in mice. **(A)** Schematic illustration of the experimental design. **(B)** Body weight changes during MPTP administration. **(C)** Rotarod test. **(D–F)** Open field test. **(G,H)** Elevated plus maze test. **(I,J)** Immunofluorescence analysis of tyrosine hydroxylase (TH)-positive neurons in the substantia nigra (SN). **(K,L)** Immunofluorescence analysis of TH-positive dopaminergic fibers in the striatum. **(M,N)** TH protein expression in the SN assessed by immunoblotting. **(O–Q)** Serum inflammatory cytokine levels (*n* = 5–6). **(O)** IL-1β; **(P)** IL-6; **(Q)** TNF-α. **(R)** Heatmap showing inflammatory cytokine expression profiles in the SN. **(S–U)** Inflammatory cytokine levels in the colon. **(S)** IL-1β; **(T)** IL-6; **(U)** TNF-α. **(V)** Heatmap showing inflammatory cytokine expression profiles in the striatum. **(W)** Representative hematoxylin and eosin (H&E) staining of colonic sections from CON and MPTP-treated mice. Green arrows indicate epithelial damage within the colonic mucosa, and black arrows indicate focal inflammatory cell infiltration. Data are presented as mean ± SEM. One-way ANOVA followed by Dunnett’s *post hoc* test: #*p* < 0.05, ##*p* < 0.01, ### *p* < 0.001 vs. MPTP group. Unpaired Student’s *t*-tests: *p < 0.05, **p < 0.01, ***p < 0.001, ****p < 0.0001 vs. MPTP group.

To recapitulate key PD features, an MPTP-induced mouse model was established (MPTP, 25 mg/kg, i.p., once daily for seven consecutive days) and behavioral performance, neuropathological changes, and inflammatory profiles were systematically evaluated (Figure 1A).

MPTP-treated mice showed reduced body-weight gain compared with controls (*p* < 0.01, Figure 1B). Motor and anxiety-like behaviors were assessed using the rotarod, open field, and elevated plus maze tests. MPTP induced motor deficits, evidenced by shorter latency to fall in rotarod test (*p* < 0.05, Figure 1C) and reduced total distance in the open field (*p* < 0.05, Figures 1D,E), with a non-significant trend toward reduced central zone exploration (*p* = 0.0993, Figure 1F). Time spent in the open arms of the elevated plus maze was also decreased (*p* < 0.01, Figures 1G,H). Dopaminergic neuronal loss in the SN was confirmed by immunofluorescence (*p* < 0.05, Figures 1I,J) and immunoblotting (*p* < 0.01, Figures 1M,N). In the striatum, TH immunofluorescence showed reduced fiber density in MPTP-treated mice (*p* < 0.0001, Figures 1K,L).

Systemic inflammatory responses were assessed in serum, SN, striatum, and colon (Figures 1O–W). Serum IL-1β (*p* < 0.001, Figure 1O), IL-6 (*p* < 0.01, Figure 1P), and TNF-α (*p* < 0.001, Figure 1Q) were elevated. In the SN, cytokine alterations were relatively limited, involving changes in IL-12p70, IL-4, and IL-10 (Figure 1R; Supplementary Figure S1A). In contrast, the striatum exhibited a broader inflammatory profile, including elevated IL-1β, IL-6, IL-12p70, IL-4, IL-17, KC, and IL-10 (Figure 1V; Supplementary Figure S1B). In the colon, IL-1β increased significantly (Figure 1S; *p* < 0.05), TNF-α showed a non-significant upward trend (Figure 1U; *p* = 0.0961) and IL-6 remained unchanged (Figure 1T). Consistent with these inflammatory changes, hematoxylin–eosin (HE) staining revealed epithelial damage and focal inflammatory infiltration in the colonic mucosa (Figure 1W).

Collectively, these findings confirm that MPTP administration recapitulates key behavioral, neuropathological, inflammatory, and intestinal alterations relevant to PD.

### 3.2 S-nicotine and R-nicotine dose-dependently ameliorate MPTP-induced motor dysfunction and restore TH expression

**Figure 2:**
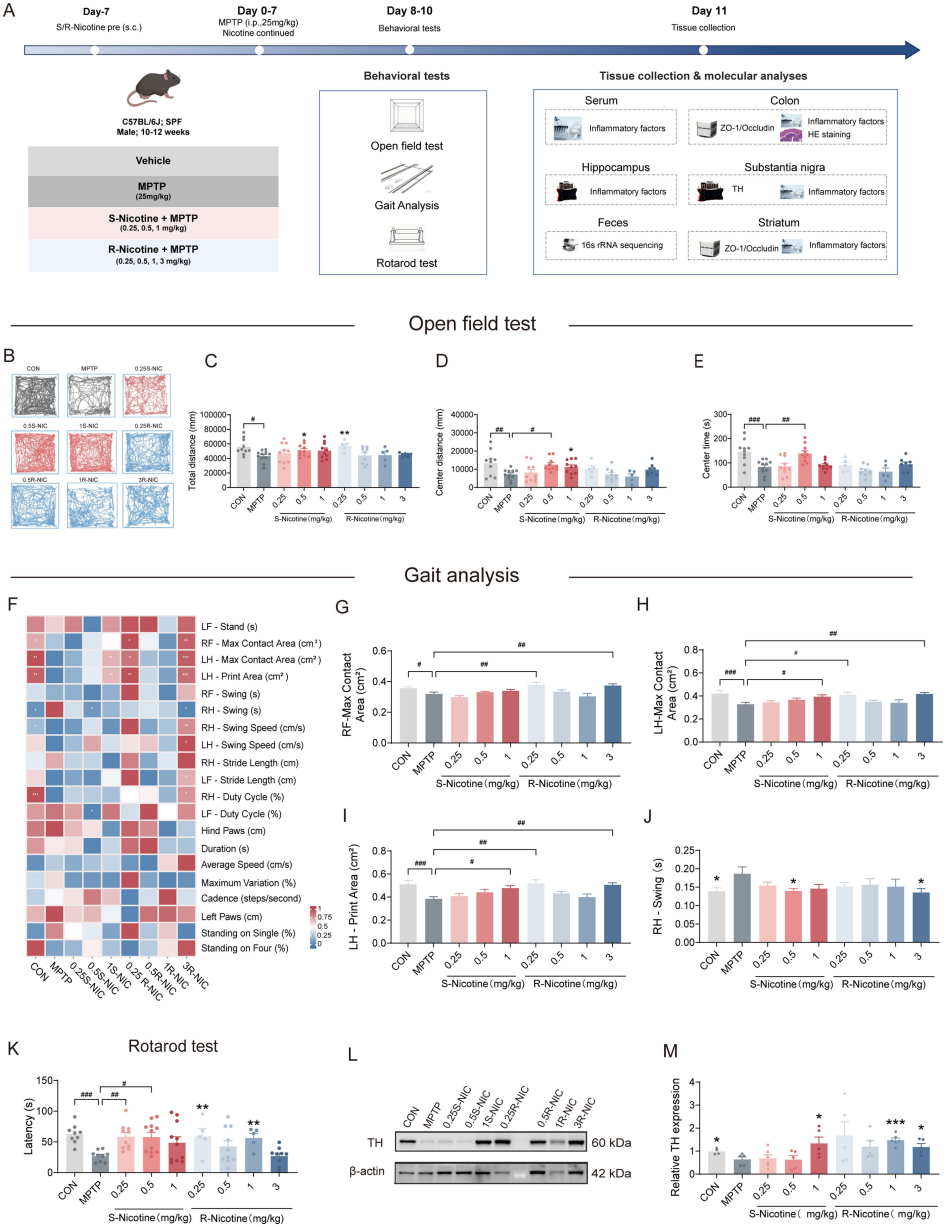
S- and R-nicotine dose-dependently ameliorate motor deficits and restore dopaminergic markers in MPTP-treated mice. **(A)** Schematic illustration of the experimental design. **(B–E)** Open field test. **(B)** Representative movement trajectories recorded over 10 min; **(C)** total distance traveled; **(D)** distance traveled in the center area; **(E)** time spent in the center area. **(F–J)** Gait analysis. **(F)** Heatmap summarizing gait parameters; **(G)** right hind (RH) paw maximum contact area; **(H)** left hind (LH) paw maximum contact area; **(I)** left hind (LH) paw print area; **(J)** right hind (RH) paw swing duration. **(K)** Rotarod test. **(L,M)** Tyrosine hydroxylase (TH) expression in the SN. **(L)** Representative immunoblots of TH and β-actin; **(M)** quantitative analysis of relative TH protein levels. Data are presented as mean ± SEM. One-way ANOVA followed by Dunnett’s *post hoc* test: # *p* < 0.05, ## *p* < 0.01, ### *p* < 0.001 vs. MPTP group. Unpaired Student’s *t*-tests: *p < 0.05, ** *p* < 0.01, *** *p* < 0.001 vs. MPTP group.

To evaluate stereoselective effects, mice received S-NIC (0.25, 0.5, or 1 mg/kg) or R-NIC (0.25, 0.5, 1, or 3 mg/kg) subcutaneously, starting 7 days before MPTP and continuing throughout treatment (Figure 2A).

In the open-field test (Figures 2B–E), S-NIC at 0.5 mg/kg significantly increased total distance, center distance (both *p* < 0.05), and center time (*p* < 0.01), whereas the 1 mg/kg increased center distance (*p* < 0.05). R-NIC exerted limited effects, only 0.25 mg/kg dose increased total distance (*p* < 0.05) and no significant improvement in center-zone exploration. Gait analysis further revealed distinct dose-dependent effects between the two enantiomers (Figures 2F–J). S-NIC at 1 mg/kg increased left hindlimb (LH) maximum contact and print area (both *p* < 0.05), and at 0.5 mg/kg reduced right hindlimb (RH) swing duration (*p* < 0.05). R-NIC improved right forelimb (RF)-Max contact area (*p* < 0.01), LH-Max contact area (*p* < 0.05), and LH print area (*p* < 0.01) at 0.25 mg/kg, and the 3 mg/kg dose further reduced RH swings (all *p* < 0.05). Intermediate R-NIC doses (0.5 and 1 mg/kg) did not produce significant gait-related effects. In the rotarod test (Figure 2K), S-NIC significantly prolonged latency to fall at all tested doses (all *p* < 0.05), while R-NIC improved performance at 0.25 and 1 mg/kg (both *p* < 0.01). Immunoblotting showed S-NIC significantly induced an increase in TH expression in the SN at 1 mg/kg (*p* < 0.05), while R-NIC showed a significant increase at 1 mg/kg (*p* < 0.001) and 3 mg/kg (*p* < 0.05). Additionally, we quantified S-nicotine and R-nicotine using UPLC-MS/MS, confirming that both S-NIC and R-NIC effectively reached the target tissues (SN, striatum, plasma, and colon, Supplementary Figures S2A–D).

Collectively, these findings indicate that both S-NIC and R-NIC attenuate MPTP-induced motor impairments and dopaminergic neuronal loss in a dose-dependent manner, with distinct stereoselective efficacy profiles.

### 3.3 S-nicotine and R-nicotine attenuate MPTP-induced neuroinflammation in serum and PD-related brain regions

**Figure 3:**
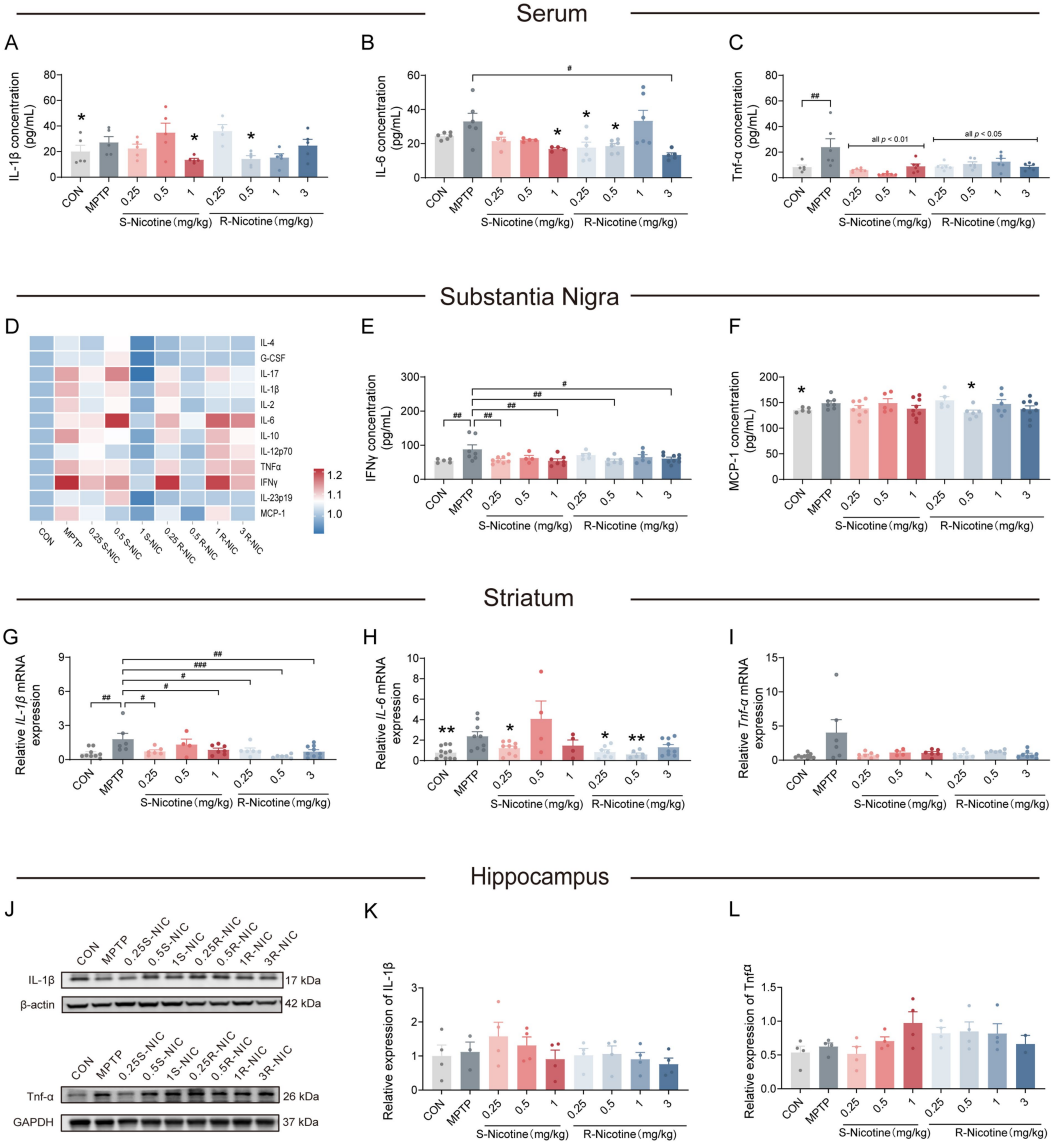
S- and R-nicotine differentially modulate systemic and central inflammatory responses in MPTP-treated mice. **(A–C)** Serum inflammatory cytokine levels. **(A)** IL-1β; **(B)** IL-6; **(C)** TNF-α. **(D–F)** Inflammatory cytokine expression in the SN. **(D)** Heatmap summarizing inflammatory cytokine profiles; **(E)** IFN-γ; **(F)** MCP-1. **(G–I)** Inflammatory cytokine mRNA expression in the striatum. **(G)** *IL-1β*; **(H)** *IL-6*; **(I)** *TNF-α*; TNF-α. **(J–L)** Inflammatory cytokine protein expression in the hippocampus. **(J)** Representative immunoblots; **(K)** quantitative analysis of IL-1β; **(L)** quantitative analysis of TNF-α. Data are presented as mean ± SEM. Statistical significance was determined by one-way ANOVA followed by Dunnett’s *post hoc* test. #*p* < 0.05, ##*p* < 0.01, ###*p* < 0.001 vs. MPTP group. Following one-way ANOVA, unpaired Student’s *t*-tests were additionally conducted to further assess the treatment effects of individual doses relative to the MPTP group. *p < 0.05, **p < 0.01 vs. MPTP group.

Given that neuroinflammation is a hallmark of PD (Houser and Tansey, 2017; Morris et al., 2024), and nicotine has been reported to exert immunomodulatory effects (Wu et al., 2025). To examine stereoselective modulation, cytokines were quantified in serum and PD-relevant brain regions (Figure 3).

In serum (Figures 3A–C), S-NIC selectively reduced IL-6 at 0.5 and 1 mg/kg (both *p* < 0.05) and TNF-α across all doses tested (all *p* < 0.05), with IL-1β significantly decreased only at 1 mg/kg (*p* < 0.05). R-NIC produced broader peripheral effects, significantly attenuating IL-1β, IL-6 and TNF-α across multiple doses (all *p* < 0.05). In the SN (Figures 3D–F), both enantiomers modulated pro-inflammatory cytokine levels in a dose-dependent manner. S-NIC significantly reduced IFNγ levels at 0.25 and 1 mg/kg (both *p* < 0.01), whereas R-NIC reduced IFNγ at 0.5 and 3 mg/kg (both *p* < 0.05) and MCP-1 at 0.5 mg/kg (*p* < 0.05). In addition, GFAP expression in the SN showed a non-significant increase following MPTP and a non-significant decrease after 3R-NIC treatment, while Iba1 levels did not differ among groups (Supplementary Figures S3A–D). In the striatum (Figures 3G–I), both S- and R-NIC significantly lowered *IL-1β* (all *p* < 0.05). *IL-6* was decreased only by S-NIC at 0.25 mg/kg (*p* < 0.05) and by R-NIC at 0.25 and 0.5 mg/kg (both *p* < 0.05), while *TNF-α* remained unchanged. Neither enantiomer significantly affected cytokine in the hippocampus (Figures 3J–L), indicating region-specific neuroinflammatory modulation.

Taken together, S-NIC and R-NIC attenuate MPTP-induced neuroinflammatory responses in a dose- and region-dependent manner.

### 3.4 Differential effects of S-nicotine and R-nicotine on MPTP-induced gut inflammation and intestinal barrier integrity

**Figure 4:**
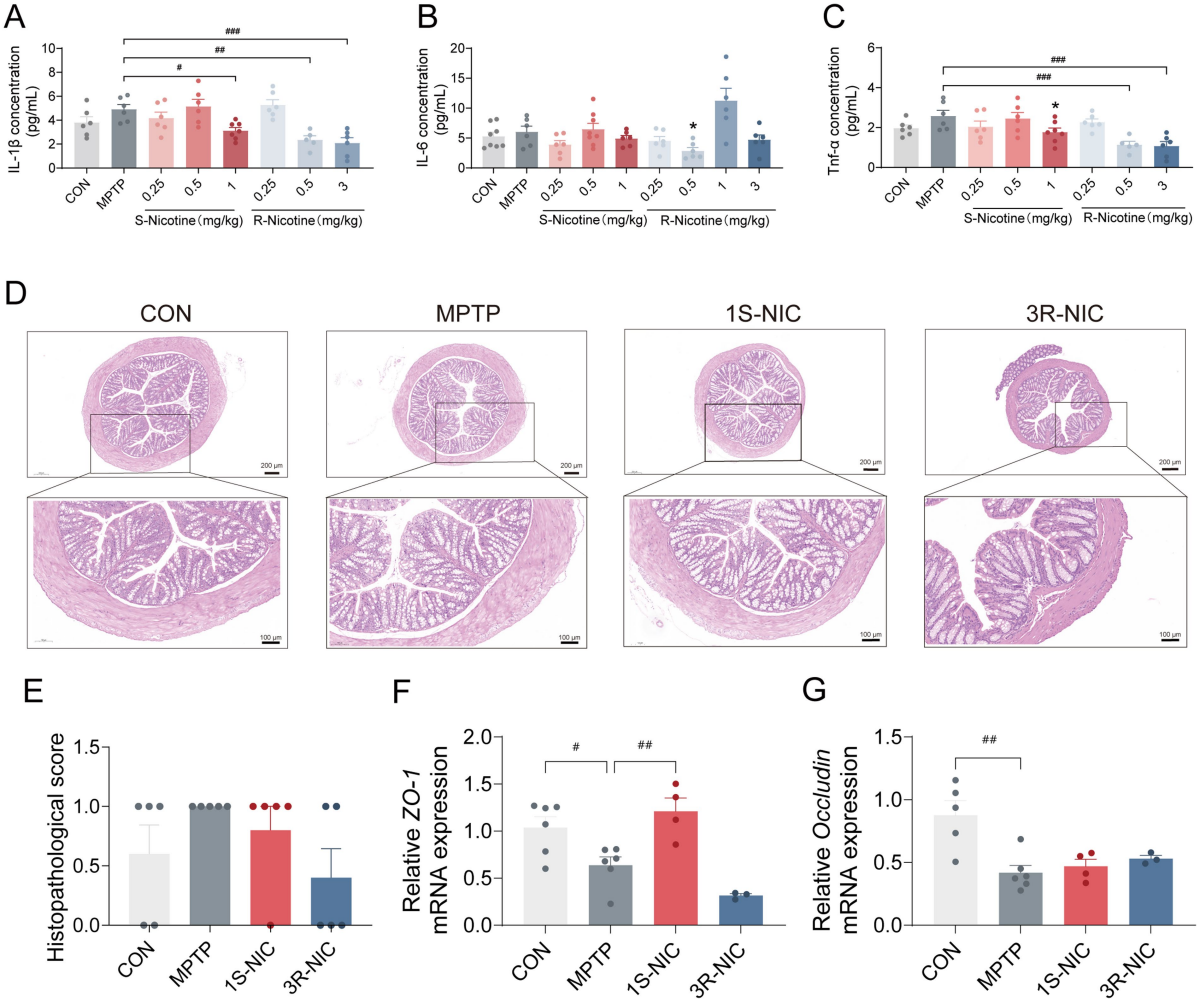
Differential effects of S- and R-nicotine on colonic inflammation and intestinal barrier integrity. **(A–C)** Protein levels of inflammatory cytokines in the colon. **(A)** IL-1β; **(B)** IL-6; **(C)** TNF-α. **(D)** Representative H&E-stained colonic sections. **(E)** Histopathological score of H&E-stained colonic sections. **(F,G)** Relative mRNA expression of tight-junction proteins in the colon. **(F)** *ZO-1* and **(G)** *Occludin*. One-way ANOVA followed by Dunnett’s *post hoc* test: #*p* < 0.05, ##*p* < 0.01, ###*p* < 0.001 vs. MPTP group. Unpaired Student’s *t*-tests: *p < 0.05, **p < 0.01, ***p < 0.001 vs. MPTP group.

Given the role of gut inflammation and barrier dysfunction in PD via the gut-brain axis (Houser and Tansey, 2017), colonic cytokines and epithelial integrity were assessed (Figure 4).

Colonic cytokine analysis revealed dose-dependent differences between the enantiomers (Figures 4A–C). S-NIC reduced IL-1β and TNF-α only at 1 mg/kg (both *p* < 0.05), whereas R-NIC displayed broader effects: 0.5 mg/kg decreased IL-1β, IL-6, and TNF-α (all *p* < 0.05), and 3 mg/kg dose suppressed IL-1β and TNF-α (both *p* < 0.001). Based on these profiles, 1 mg/kg S-NIC and 3 mg/kg R-NIC were selected for subsequent intestinal barrier analyses. Histological examination showed that overall colonic architecture was largely preserved across all groups. No inflammatory cell infiltration, epithelial necrosis, or glandular depletion was observed. Semi-quantitative scoring of epithelial structural integrity (0–3 scale) revealed only mild alterations (scores 0–1) in all groups, with no significant differences detected among groups (Figures 4D,E). Tight-junction analysis further supported differential barrier effects: S-NIC significantly restored *ZO-1* mRNA (*p* < 0.01, Figure 4F) but not *Occludin* mRNA (Figure 4G), while R-NIC failed to significantly restore either *ZO-1* or *Occludin* mRNA, suggesting differential effects on epithelial barrier integrity.

Collectively, both nicotine enantiomers reduced colonic inflammation, while S-NIC exhibited a more pronounced protective effect on intestinal barrier integrity at its optimal dose.

### 3.5 Differential effects of S-nicotine and R-nicotine on gut microbiota diversity

**Figure 5:**
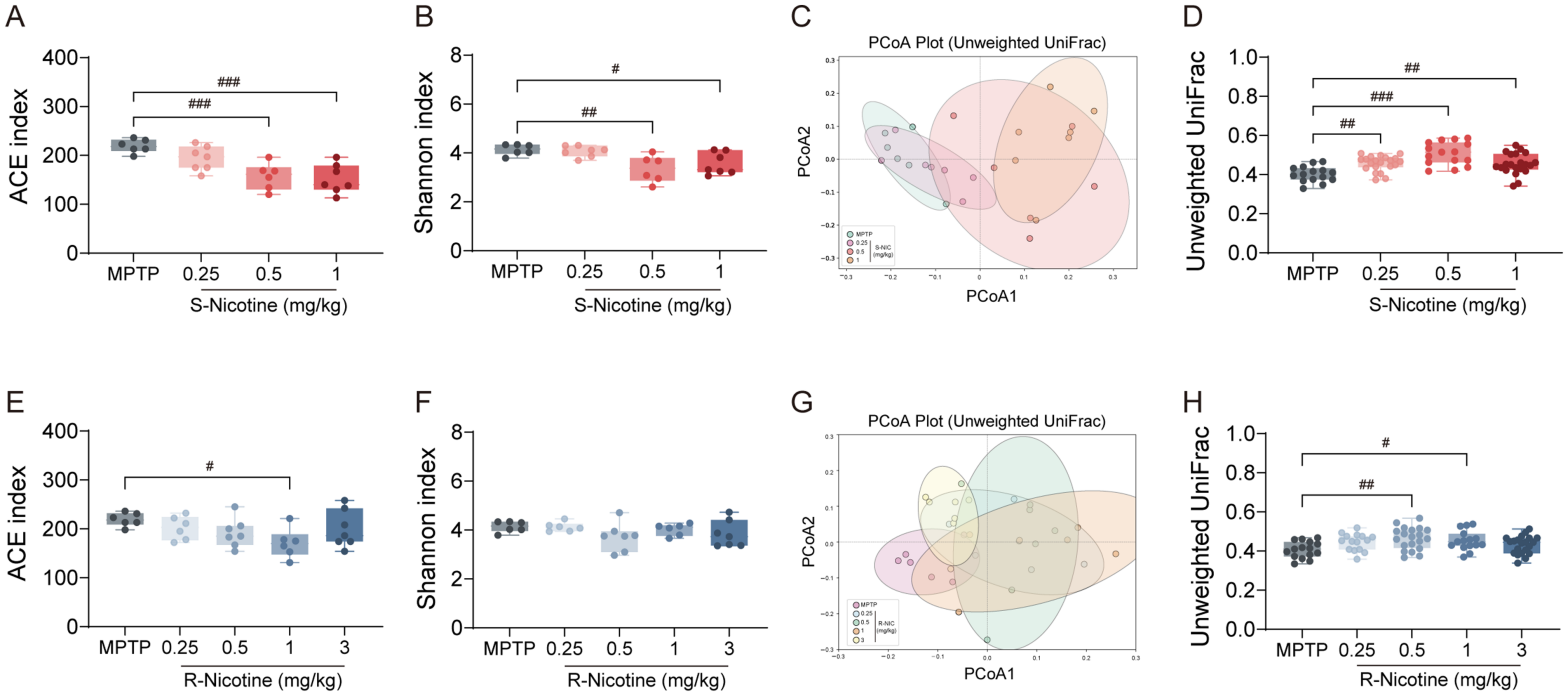
Differential effects of S- and R-nicotine on gut microbiota diversity in MPTP-treated mice. **(A–D)** Gut microbiota diversity analysis in the S-NIC-treated mice. **(A,B)** α-diversity indices, including **(A)** ACE and **(B)** Shannon indices. **(C)** PCoA plot based on unweighted UniFrac distances. **(D)** Quantification of unweighted UniFrac distances relative to the MPTP group. **(E–H)** Gut microbiota diversity analysis in R-NIC-treated mice. **(E,F)** α-diversity indices, including **(E)** ACE and **(F)** Shannon indices. **(G)** PCoA plot based on unweighted UniFrac distances. **(H)** Quantification of unweighted UniFrac distances relative to the MPTP group. Data are presented as mean ± SEM. Statistical significance was determined by one-way ANOVA followed by Dunnett’s *post hoc* test. #*p* < 0.05, ## *p* < 0.01, ### *p* < 0.001 vs. MPTP group.

Given the close association between PD and gut microbiota dysbiosis (Wang et al., 2021; Zheng et al., 2021), 16S rRNA sequencing was performed to determine whether nicotine enantiomers differentially affect gut microbial composition (Figure 5).

α-diversity analysis revealed that S-NIC reduced microbial richness and diversity in a dose-dependent manner (Figures 5A,B; Supplementary Figures S4A–C). Specifically, ACE (Figure 5A), Shannon (Figure 5B), Sobs (Supplementary Figure S4A), and Chao (Supplementary Figure S4B) indices were significantly reduced at 0.5 and 1 mg/kg, while Simpson index (Supplementary Figure S4C) showed a significant reduction only at 1 mg/kg, indicating a contraction of microbial diversity at higher doses. In contrast, R-NIC exerted milder effects, with ACE (Figure 5E), Sobs (Supplementary Figure S3D), and Chao (Supplementary Figure S4E) reduced only at 1 mg/kg, while Shannon (Figure 5F) and Simpson (Supplementary Figure S4F) indices remained unchanged.

β-Diversity analysis using unweighted UniFrac distances revealed distinct community shifts (Figures 5C,D,G,H). Principal Coordinates Analysis (PCoA) showed S-NIC-treated groups progressively shifted away from the MPTP cluster in a dose-dependent manner (Figure 5C), with significantly increased UniFrac distances at all doses (all *p* < 0.01, Figure 5D). R-NIC induced modest alterations. PCoA showed partial separation from MPTP (Figure 5G) and unweighted UniFrac distance increased at 0.5 and 1 mg/kg, but without a clear dose-dependent pattern (Figure 5H).

Overall, these results indicate that S-NIC exerts a stronger and dose-dependent modulatory effect on gut microbial diversity and community structure, whereas R-NIC induces moderate and less coordinated alterations.

### 3.6 S- and R-nicotine differentially modulate gut microbial community structure

**Figure 6:**
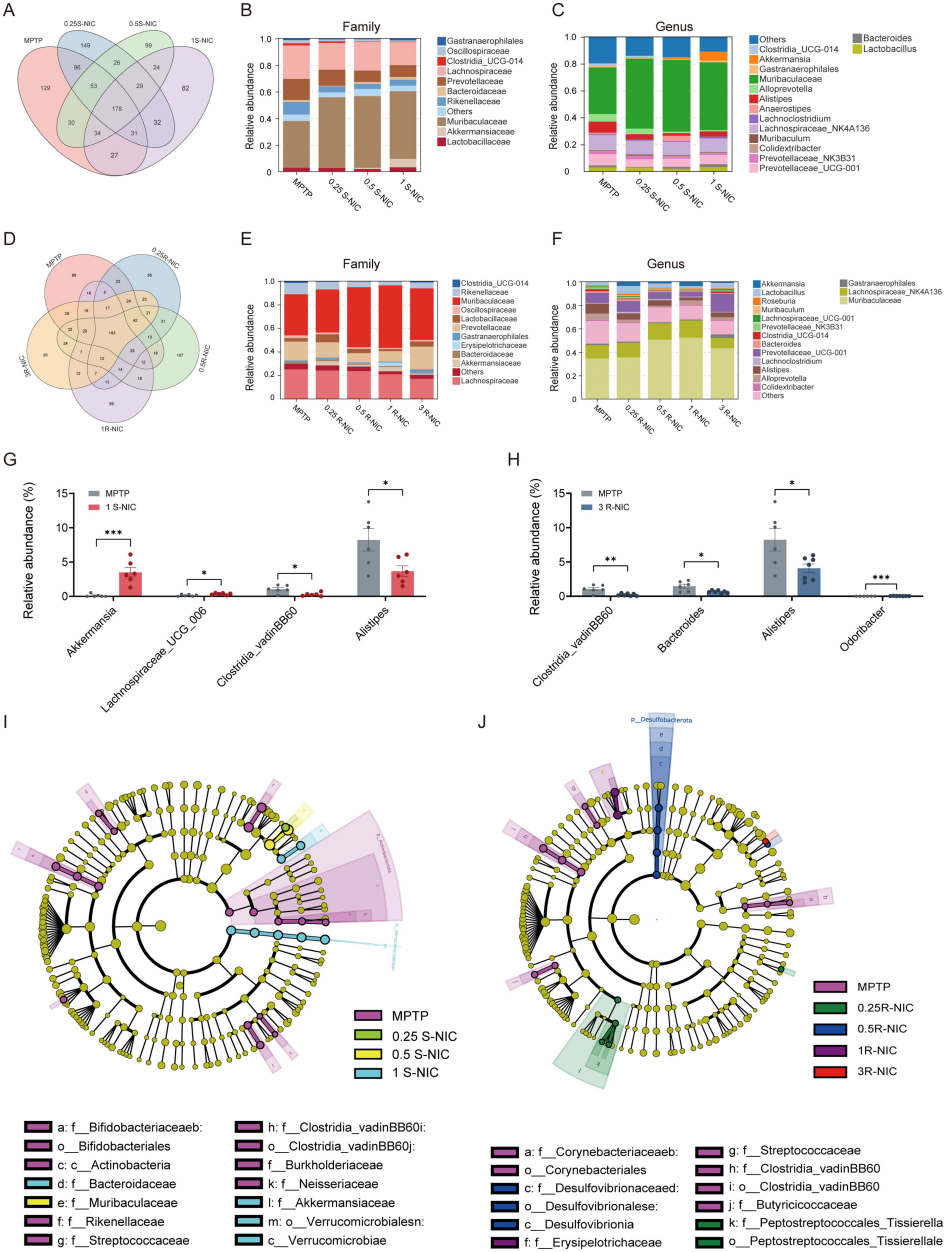
S- and R-nicotine differentially alter gut microbial composition at the OTU, family, and genus levels. **(A,D)** Venn diagrams showing shared and unique operational taxonomic units (OTUs) among experimental groups treated with S-NIC **(A)** or R-NIC (D). **(B,E)** Relative abundance of gut microbiota at the family level in S-NIC-treated **(B)** and R-NIC-treated **(E)** groups. **(C,F)** Relative abundance of gut microbiota at the genus level in S-NIC-treated **(C)** and R-NIC-treated **(F)** groups. **(G,H)** Relative abundance of selected PD-associated genera in S-NIC-treated (**G**; *Akkermansia*, *Lachnospiraceae_UCG_006*, *Clostridia_vadinBB60*_group, and *Alistipes*) and R-NIC-treated mice (H; *Clostridia_vadinBB60*_group, *Bacteroides*, *Alistipes*, and *Odoribacter*). **(I,J)** Linear discriminant analysis effect size (LEfSe) identifying differentially enriched taxa in S-NIC-treated **(I)** and R-NIC-treated **(J)** groups. Data are presented as mean ± SEM. Statistical significance was determined using an unpaired Student’s *t*-test. *p < 0.05, **p < 0.01, ***p < 0.001 vs. MPTP Group.

To further characterize the effects of nicotine enantiomers on gut microbiota composition, OTU-level analyses were first performed (Figures 6A,D), followed by taxonomic profiling at the family and genus levels (Figures 6B,C,E,F).

Venn diagram analysis revealed that 178 OTUs were shared among all S-NIC-treated groups, whereas the MPTP group exhibited a substantially higher number of unique OTUs (Figure 6A). Notably, S-NIC treatment markedly reduced the number of MPTP-specific OTUs across increasing doses, suggesting a partial restructuring of microbial community membership. In contrast, 183 OTUs were shared between the MPTP and R-NIC-treated groups, with fewer shared OTUs and a higher proportion of dose-specific OTUs across R-NIC treatments (Figure 6D), indicating higher intergroup variability in microbial community membership.

At the family level, both nicotine enantiomers modulated gut microbiota composition relative to the MPTP group, but with distinct magnitudes and patterns. S-NIC induced a pronounced and dose-dependent restructuring, characterized by marked enrichment of Muribaculaceae and Akkermansiaceae, accompanied by a progressive reduction in Lachnospiraceae and related anaerobic fermentative families (Figure 6B). In contrast, R-NIC produced comparatively modest alterations. Muribaculaceae showed a moderate increase at intermediate doses, while Lachnospiraceae gradually decreased with increasing dose. However, most other families exhibited only minor fluctuations without a consistent dose–response relationship (Figure 6E).

At the genus level, S-NIC markedly enriched Muribaculaceae-associated genera and *Akkermansia*, particularly at 1 mg/kg (Figure 6C). Targeted analysis of PD-related genera further confirmed that *Akkermansia* (*p* < 0.001) and *Lachnospiraceae_UCG_006* (*p* < 0.05) were significantly increased, whereas *Clostridia_vadinBB60_group*, *Alistipes*, *Clostridia_UCG_014*, *Cutibacterium*, and *Staphylococcus* (all *p* < 0.05) were significantly decreased in the 1 mg/kg S-NIC group compared with the MPTP group (Figure 6G; Supplementary Figure S5A). In contrast, R-NIC induced more subtle genus-level alterations (Figures 6F–H and Supplementary Figure S5B), with reductions in *Clostridia_vadinBB60_group*, *Bacteroides*, *Alistipes*, *Corynebacterium*, *Prevotella*, and *Bilophila*, accompanied by increase in *Odoribacter* (all *p* < 0.05).

LEfSe analysis further revealed enantiomer-specific microbiota signatures (Figures 6I,J). The MPTP group was characterized by enrichment of taxa such as *Clostridia_vadinBB60_group*, Streptococcaceae, and Rikenellaceae. S-NIC selectively enriched phylogenetically coherent taxa, particularly Muribaculaceae, Akkermansiaceae (including *Akkermansia*), and members of the phylum Verrucomicrobia, indicating substantial community restructuring (Figure 6I; Supplementary Figure S5C). In contrast, R-NIC treatment enriched fewer and more phylogenetically dispersed taxa, such as Desulfovibrionaceae and Peptostreptococcales_Tissierellales (Figure 6J; Supplementary Figure S5D), without inducing a dominant phylogenetic shift.

Overall, these findings demonstrate that S-NIC induces a more coherent and disease-relevant remodeling of the gut microbiota, characterized by enrichment of Akkermansiaceae (notably *Akkermansia*) and Muribaculaceae, and concomitant reductions in *Clostridia_vadinBB60_group*, *Alistipes*, and Lachnospiraceae-related taxa, whereas RNIC primarily reduced *Clostridia_vadinBB60_group*, *Bacteroides*, and *Alistipes*, while increasing *Odoribacter* abundance.

### 3.7 Associations between gut microbiota and PD-related behavioral, inflammatory, and intestinal parameters

**Figure 7:**
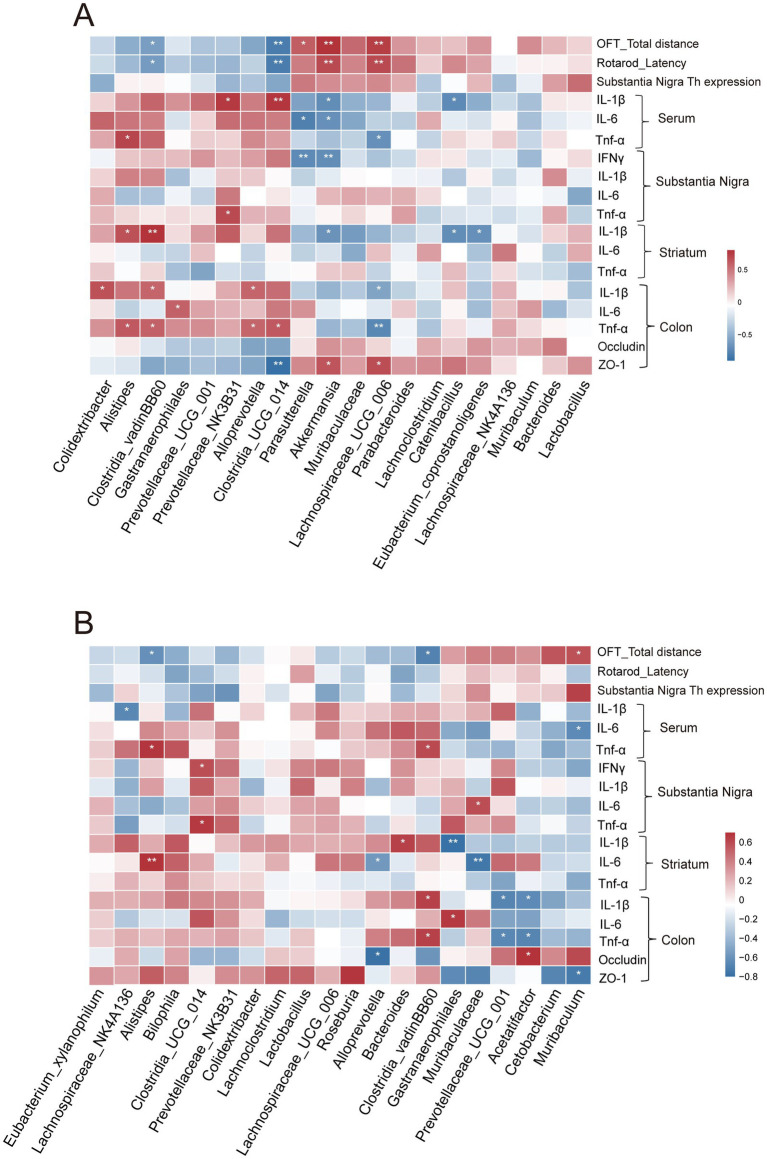
Spearman correlation analysis between gut microbiota and PD-related parameters. **(A,B)** Spearman correlation heatmaps showing associations between the top 20 genera and PD-related behavioral, inflammatory, and intestinal barrier indicators in S-NIC-treated **(A)** and R-NIC-treated mice **(B)**. Color intensity indicates the strength of the correlation. *p < 0.05, **p < 0.01.

To further explore potential associations between gut microbiota alterations and PD-related phenotypes, Spearman correlation analyses were performed between genus-level bacterial abundances and behavioral performance, inflammatory markers, and intestinal barrier indicators in mice treated with S-NIC or R-NIC (Figures 7A,B; Supplementary Figures S6A–G).

In the S-NIC-treated mice (1 mg/kg, Figure 7A; Supplementary Figures S6A–G), the relative abundance of *Akkermansia* and *Lachnospiraceae_UCG_006* was positively correlated with motor performance indices, including total distance in open-field test (OFT) and rotarod latency, as well as TH expression in the SN (all *p* < 0.01). These genera were also negatively correlated with pro-inflammatory cytokines (IL-1β, IL-6, and TNF-α) in serum, SN, and striatum (all *p* < 0.05). Additionally, the relative abundance of *Akkermansia* and *Lachnospiraceae_UCG_006* showed significant positive correlations with intestinal barrier markers *ZO-1* (both *p* < 0.05). Conversely, the relative abundances of *Clostridia_vadinBB60_group*, *Alistipes* and *Clostridia_UCG_014*, were negatively correlated with motor performance (all *p* < 0.05), while positively correlated with inflammatory cytokines levels across both central and peripheral tissues (all *p* < 0.05). Moreover, *Clostridia_UCG_014* abundance was also negatively correlated with intestinal barrier integrity indicators (*p* < 0.01). In contrast, the R-NIC-treated mice (3 mg/kg, Figure 7B), the relative abundances of *Clostridia_vadinBB60* group and *Alistipes*, showed significantly positive correlations with inflammatory cytokines levels in both peripheral and central compartments (all *p* < 0.05), whereas correlations with motor performance or intestinal barrier markers were limited or absent.

Collectively, S- and R-NIC exhibited both shared and enantiomer-specific effects on the gut microbiota. While both reduced PD-associated taxa such as *Clostridia_vadinBB60_group* and *Alistipes*, S-nicotine uniquely enriched beneficial genera including *Akkermansia* that were closely associated with improved motor performance, reduced inflammation, and enhanced intestinal barrier integrity, whereas R-nicotine induced more limited and less coordinated microbial changes.

## 4 Discussion

**Figure 8:**
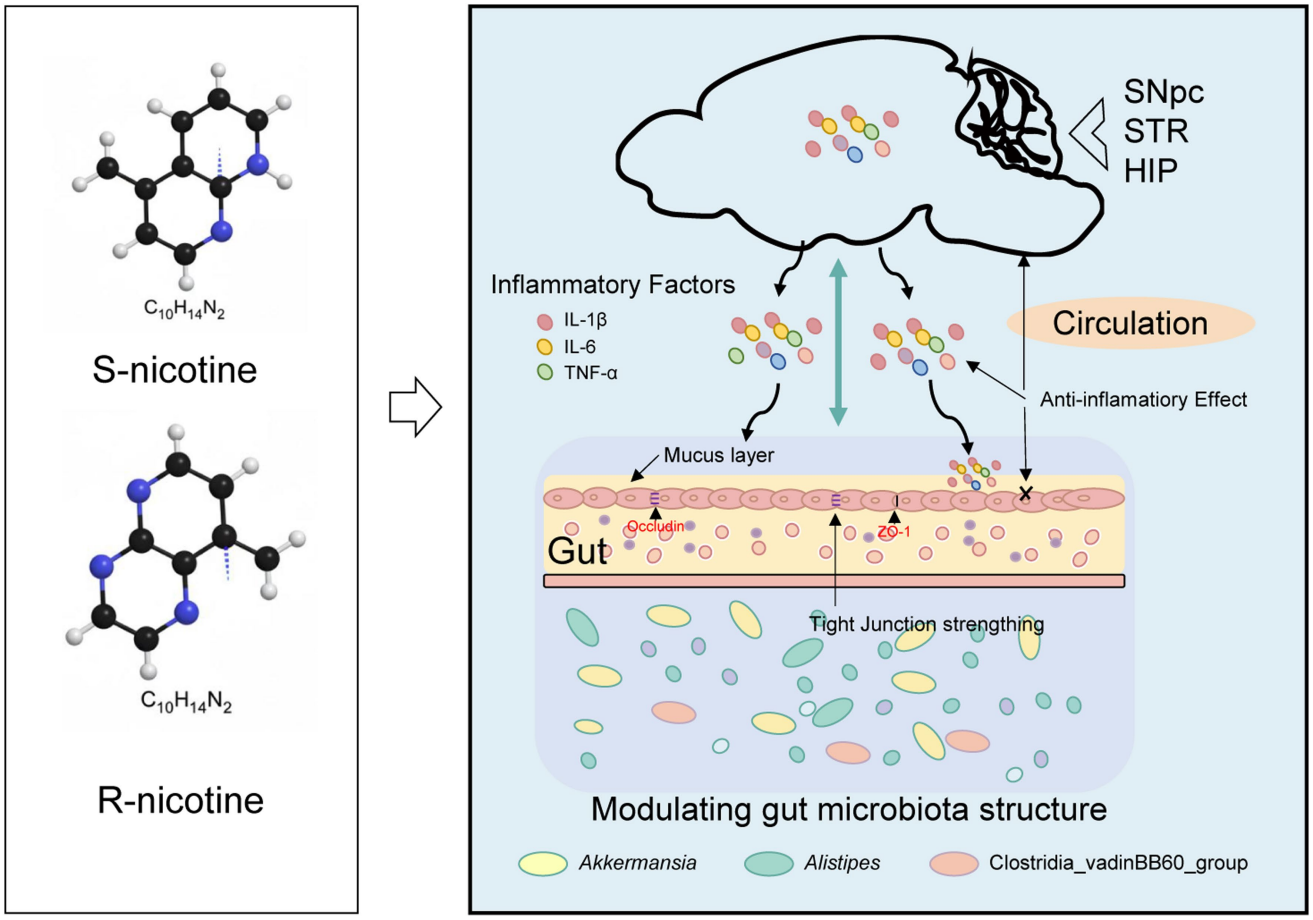
Stereoselective effects and mechanisms of nicotine enantiomers on the gut-brain axis in MPTP-induced PD mice.

In this study, we systematically compared the dose-dependent and stereoselective effects of S-NIC and R-NIC in an MPTP-induced PD mouse model, focusing on neuroprotection, inflammation, intestinal pathology, and gut microbiota remodeling (Figure 8). Both enantiomers provided protective effects, but S-NIC and R-NIC displayed distinct efficacy profiles, optimal dose ranges, and differential modulation of the microbiota-gut-brain axis, highlighting the importance of stereochemistry in nicotine pharmacology.

Consistent with epidemiological observations reports linking tobacco use to reduced PD risk, nicotine has been proposed as a potential neuroprotective agent in PD (Hernán et al., 2002; Ritz et al., 2007; Mappin-Kasirer et al., 2020). However, such associations do not establish causality and may be confounded by behavioral or environmental factors. Moreover, tobacco exposure involves complex toxic mixtures, and its relationship with PD cannot be attributed to nicotine alone, highlighting the need to distinguish epidemiological correlations from controlled pharmacological evidence. Experimental studies show that nicotine attenuates dopaminergic neurodegeneration, suppresses neuroinflammation, and modulates synaptic plasticity via nAChRs, especially α4β2 and α7 subtypes (Liu et al., 2012; Kumari et al., 2025; Lin et al., 2025; Wu et al., 2025). Here, both S- and R-NIC improved motor performance and partially restored TH expression in the SN, with S-NIC effective at lower doses. This stereoselective pharmacodynamics aligns with known higher receptor affinity and efficacy of S-NIC (Yang et al., 2019; Jiang et al., 2024). However, it is critical to acknowledge nicotine’s well-established adverse effects, including addictive (Benowitz, 2010), cardiovascular, respiratory (Munzel et al., 2025), and neurodevelopmental liabilities (Little et al., 2021). In addition, its effects can be dose- and context-dependent, with potential pro-inflammatory or neurotoxic outcomes under certain conditions. These considerations emphasize that nicotine is not inherently neuroprotective, but exhibits a context-dependent therapeutic window.

Neuroinflammation is a key driver of PD progression (Hirsch and Hunot, 2009; Ransohoff, 2016). Both enantiomers attenuated MPTP-induced inflammatory responses in central and peripheral compartments, but with different patterns. S-NIC displayed region-specific anti-inflammatory effects, whereas R-NIC broadly suppressed cytokine expression across multiple compartments, suggesting differential engagement of nAChR subtypes on glial and immune cells (Liu et al., 2012; Soares et al., 2024; Wu et al., 2025). Consistent with previous reports that MPTP induces astrocyte and microglial activation (Sun et al., 2018), while nicotinic signaling can exert anti-inflammatory effects on glial cells (Soares et al., 2024), we further assessed GFAP and Iba1 protein levels in the SN by Western blot. MPTP induced a trend toward increased GFAP expression, while 3R-NIC showed a tendency to attenuate this effect, whereas Iba1 levels remained unchanged across groups. These findings suggest that astrocytic responses may be modestly modulated under our conditions, while microglial alterations are not prominently reflected at the total protein level. However, given that glial activation involves dynamic morphological changes that cannot be captured by bulk protein analysis, these results should be interpreted with caution. The lack of significant cytokine changes in the hippocampus across all treatment groups may reflect the relative resistance of this region to MPTP-induced neuroinflammation or differential nAChR subtype distribution, warranting further investigation.

Beyond neuroinflammation, nicotine enantiomers also differentially modulate gut inflammation, intestinal barrier integrity, and gut microbiota composition. Gastrointestinal dysfunction and microbial dysbiosis are increasingly recognized as early and integral components of PD pathogenesis (Forsyth et al., 2011; Scheperjans et al., 2014; Sampson et al., 2016; Lai et al., 2018). Nicotine has been reported to exert gut anti-inflammatory effects by activating the cholinergic anti-inflammatory pathway and suppressing pro-inflammatory cytokines in experimental colitis models (Lakhan and Kirchgessner, 2011; Nakajima et al., 2021). In our model, S-NIC better preserved intestinal barrier integrity, restoring *ZO-1* expression, whereas R-NIC reduced inflammation but did not affect tight-junction markers, suggesting barrier-protective mechanisms beyond simple anti-inflammatory action. Notably, no overt colonic histopathology was observed across groups, suggesting that the MPTP model primarily affects central nervous system pathology rather than inducing robust intestinal structural damage. The lack of significant histological differences also indicates that the effects of nicotine may be more functional or microbiota-related rather than structural at the tissue level.

Gut microbiota analysis further revealed pronounced enantiomer-specific effects. At the community level, S-NIC promoted coherent, disease-relevant remodeling, whereas R-NIC produced less coordinated changes. Reduced OTU overlap in R-NIC groups, despite apparent restructuring, may reflect microbial instability rather than therapeutic correction (Lozupone et al., 2012; Sommer et al., 2017). Taxonomic analyses further support this distinction. S-NIC enriched Akkermansiaceae (notably *Akkermansia muciniphila*) and Lachnospiraceae genera, while reducing PD-associated taxa such as *Clostridia_vadinBB60_group* and *Alistipes*. *Akkermansia* enhances intestinal barrier function, thickens the mucus layer, and suppresses systemic inflammation (Mo et al., 2024; Panzetta and Valdivia, 2024). In both PD patients and animal models, decreased *Akkermansia* abundance has been associated with increased intestinal permeability and elevated neuroinflammation (Qiao et al., 2024; Zhao et al., 2024; Xu et al., 2025). In our study, S-NIC-induced *Akkermansia* enrichment correlated with improved motor behavior, SN TH expression, colonic ZO-1 levels, and reduced inflammatory cytokines, supporting coordinated gut-brain protective effects. Muribaculaceae, also enriched by S-NIC, are involved in complex polysaccharide degradation and short-chain fatty acid production, potentially contributing to gut health (Zhu et al., 2024). *Lachnospiraceae_UCG_006*, a butyrate-producing taxon, contributes to colonic epithelial health and immune modulation (Nagarajan et al., 2023; Yang et al., 2025). S-NIC increased its abundance, correlating with improved behavior, reduced inflammation, and restored barrier function, although it remained less abundant than dominant taxa (Romano et al., 2021). Conversely, S-NIC reduced *Alistipes* and *Clostridia_vadinBB60_group*, both linked to pro-inflammatory states and dysbiosis (Fu et al., 2024; Zhao et al., 2024; Chen et al., 2025). Overall, S-NIC selectively enriches barrier-supportive, anti-inflammatory microbes while suppressing harmful taxa, promoting microbiota-gut-brain axis restoration and neuroprotection.

R-NIC induced more limited microbial shifts. Although it reduced inflammation-associated genera, it failed to enrich barrier-supportive taxa consistently. Notably, R-NIC increased *Odoribacter*, a short-chain fatty acid-producing genus whose role in PD remains unclear (Li et al., 2025a). Despite potential anti-inflammatory effects of butyrate, increased *Odoribacter* may reflect dysbiosis (Alshehri et al., 2021; Li et al., 2025a), which may underlie R-NIC’s limited capacity to restore intestinal barrier integrity or fully coordinate gut-brain interactions. Future functional studies are needed to determine whether *Odoribacter* plays a protective or detrimental role in the context of PD.

Several limitations should be noted. First, the MPTP model is an acute toxin-based paradigm that does not fully capture chronic, progressive PD pathology or α-synuclein-related Lewy body formation (Wichmann et al., 2025). Second, our study focused on short-term outcomes, leaving the long-term efficacy and safety of nicotine enantiomers untested. Third, while correlative analyses implicate the gut microbiota, causality remains unproven and requires microbiota depletion or fecal microbiota transplantation. Fourth, neuroinflammatory assessment in this study relied primarily on cytokine measurements and bulk protein analysis, without cell-type-specific or morphology-based characterization of glial activation (e.g., microglial activation and astrocyte reactivity), which limits mechanistic interpretation. Additional limitations include small sample sizes, potential sex-related variability, and lack of systemic assessment such as cardiovascular effects. Importantly, although nicotine enantiomers showed coordinated neuroprotective and gut-brain effects, their translational application is limited by well-established systemic toxicity and addictive liability. Therefore, future therapeutic strategies should prioritize selective nicotinic receptor targeting or peripheral gut-oriented modulation rather than direct nicotine administration.

## 5 Conclusion

In conclusion, S- and R-NIC exert distinct, dose-dependent, stereoselective effects on dopaminergic neurodegeneration, neuroinflammation, intestinal pathology, and gut microbiota composition. S-NIC demonstrates superior efficacy at lower doses, coordinating barrier integrity, microbiota-gut-brain remodeling, and anti-inflammatory responses. R-NIC reduces inflammation but has a narrower therapeutic window and less integrated microbiota modulation. These findings underscore stereochemistry as a critical determinant in nicotine pharmacology and highlight the microbiota-gut-brain axis as a target for chiral interventions. S-NIC emerges as a promising candidate for neuroprotective nicotine derivatives, with translational potential for therapies leveraging both central and peripheral mechanisms. Future studies should further investigate the long-term efficacy, safety, and mechanistic specificity of nicotine enantiomers in chronic and α-synuclein-based PD models, as well as explore receptor subtype-selective and peripherally restricted analogs to improve therapeutic specificity. From a clinical perspective, these findings support cholinergic and microbiota-gut-brain axis modulation as a potential strategy for early intervention in Parkinson’s disease, particularly for non-motor and prodromal gastrointestinal symptoms.

## Data Availability

The datasets presented in this study are available in online repositories. The raw 16S rRNA sequencing data have been deposited in the NCBI BioProject database under accession number PRJNA1398440 (https://www.ncbi.nlm.nih.gov/bioproject/PRJNA1398440). All other data supporting the findings of this study are available from the corresponding author upon reasonable request.
