# Supplementary material for: Stereoselective effects of nicotine enantiomers on the gut-brain axis and neuroinflammation in a mouse model of Parkinson’s disease
Source: Front Aging Neurosci. 2026 May 13;18:1823372. doi: 10.3389/fnagi.2026.1823372 (PMC13212218; doi:10.3389/fnagi.2026.1823372)
Supplement: Supplementary file 1 [file Data_Sheet_1.DOCX]

Supplementary Material

## Supplementary Figures


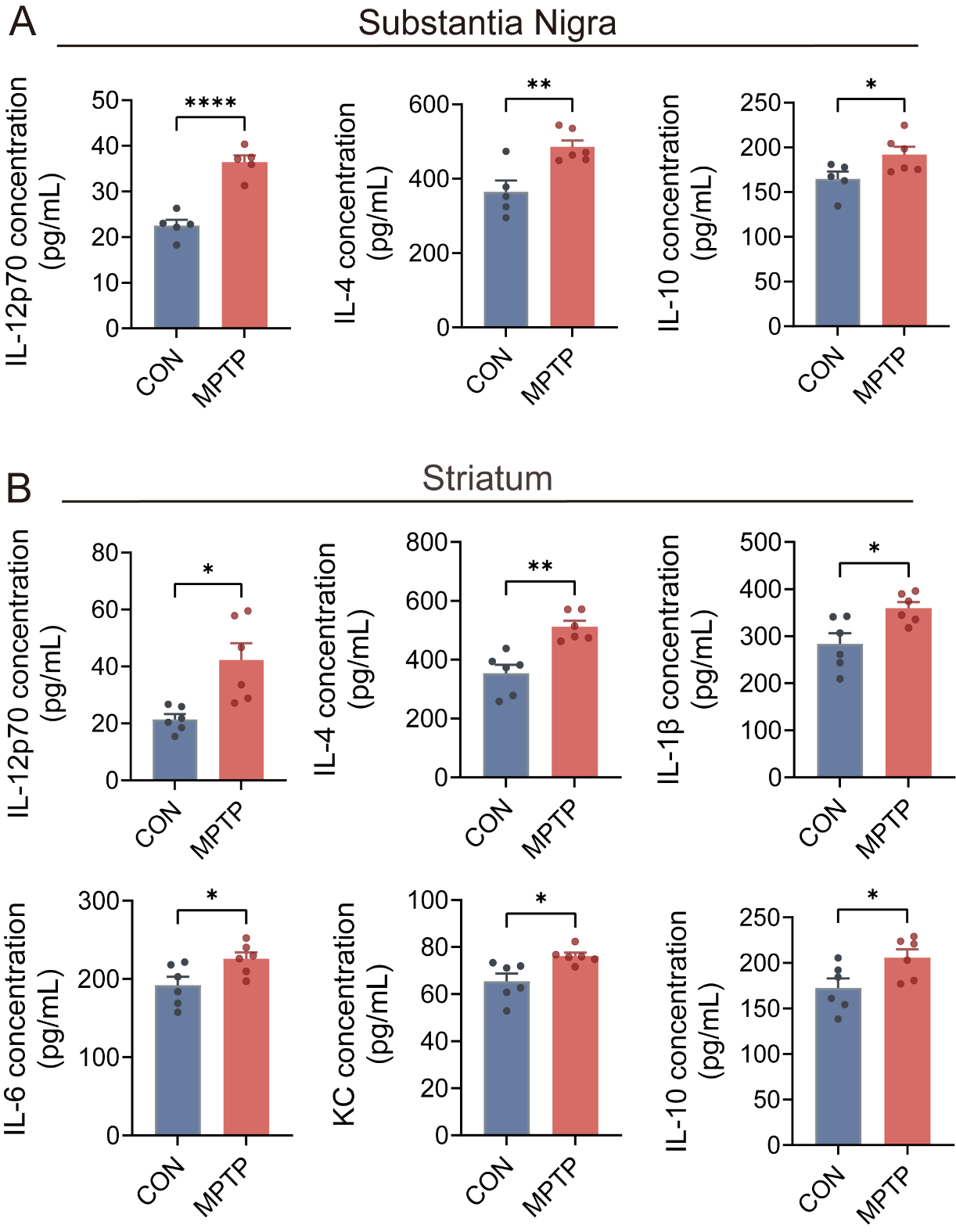


**Supplementary Figure 1.** MPTP Treatment Induces Severe Neuroinflammation in Mice. (A) Inflammatory cytokine protein expression in the Substantia Nigra (SN). Levels of IL-12p70, IL-4, and IL-10 were measured to evaluate the neuroinflammatory response. (B) Inflammatory cytokine protein expression in the Striatum. Comprehensive analysis of cytokine profiles including IL-12p70, IL-4, IL-1β, IL-6, KC, and IL-10. Data are presented as mean ± SEM; n = 5-6 per group. The differences between the CON group and the MPTP group were analyzed by unpaired student’s *t*-tests, **p* < 0.05, ** *p* < 0.01, *** *p* < 0.001 vs. MPTP group.


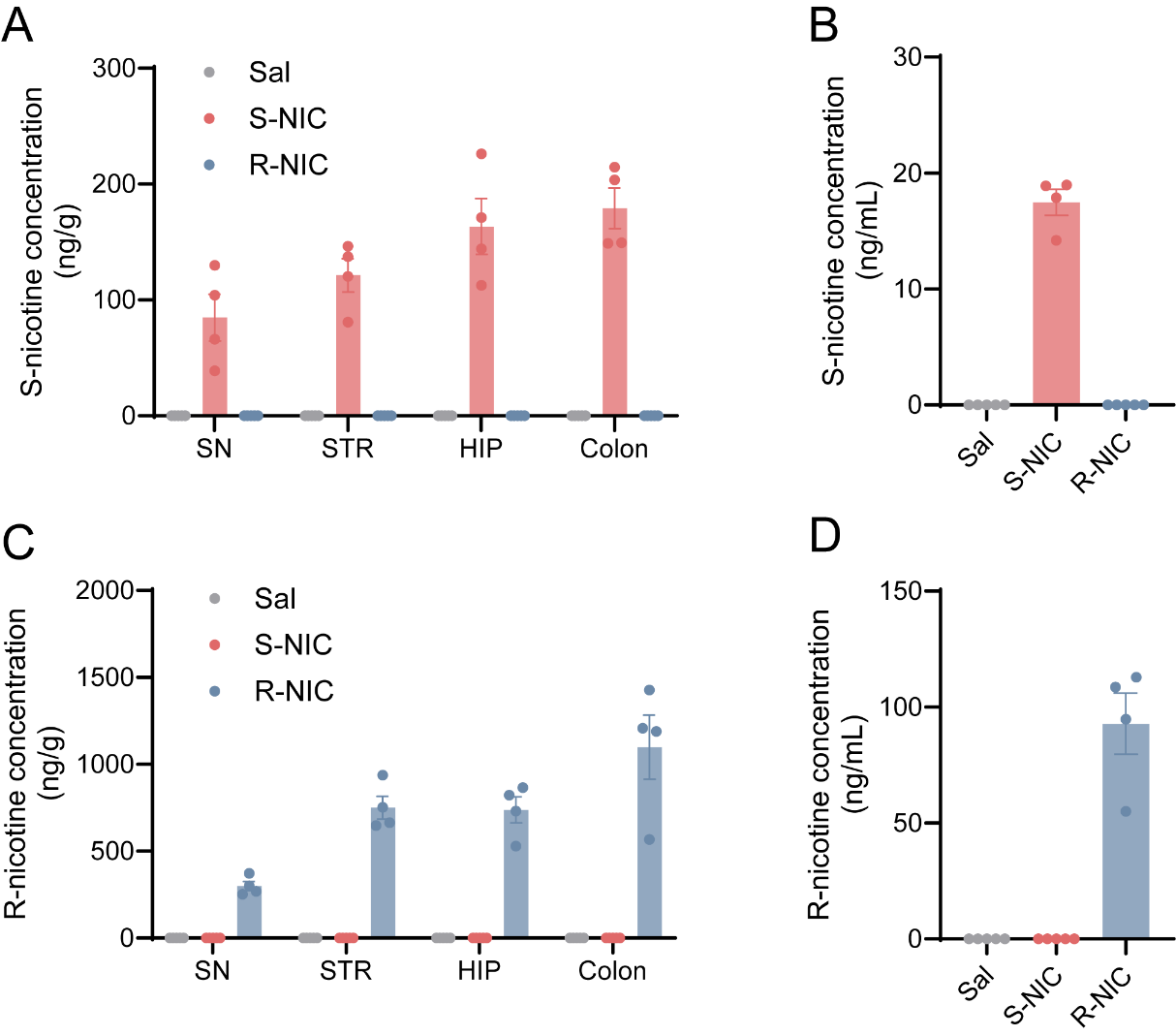


**Supplementary Figure 2.** Tissue levels of S-Nicotine and R-Nicotine in Mice. (A) Tissue levels of S-nicotine in the substantia nigra (SN), striatum (STR), hippocampus (HIP), and colon following systemic administration. (B) Plasma levels of S-nicotine. (C) Tissue levels of R-nicotine in the SN, STR, HIP, and colon following systemic administration. (D) Plasma levels of R-nicotine. Data are presented as mean ± SEM; n = 4-5 per group.


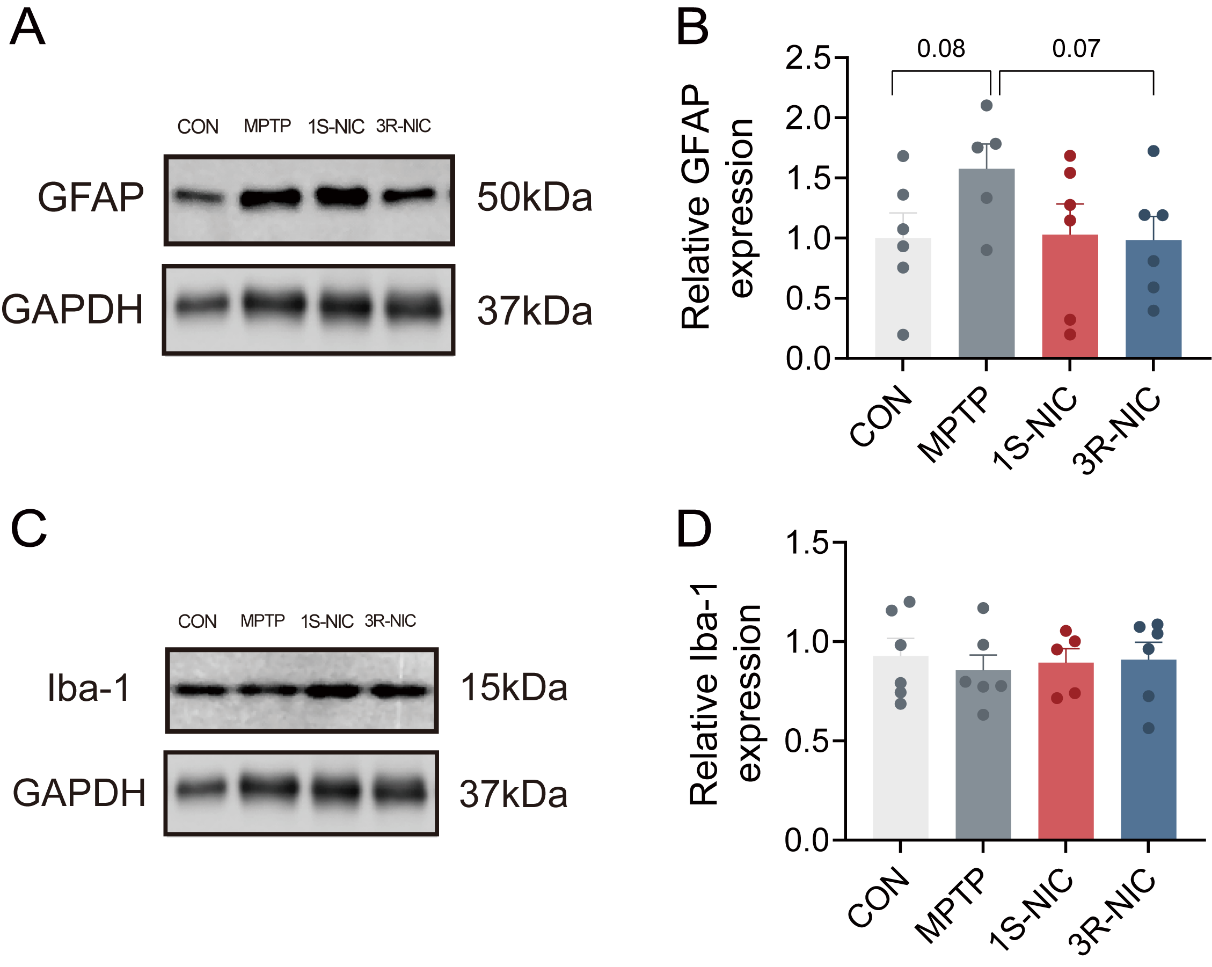


**Supplementary Figure 3.** Effects of S-Nic and R-Nic on the protein expression levels of GFAP and Iba1 in the substantia nigra. (A) Representative immunoblots; (K) quantitative analysis of GFAP. (C) Representative immunoblots; (D) quantitative analysis of Iba1.


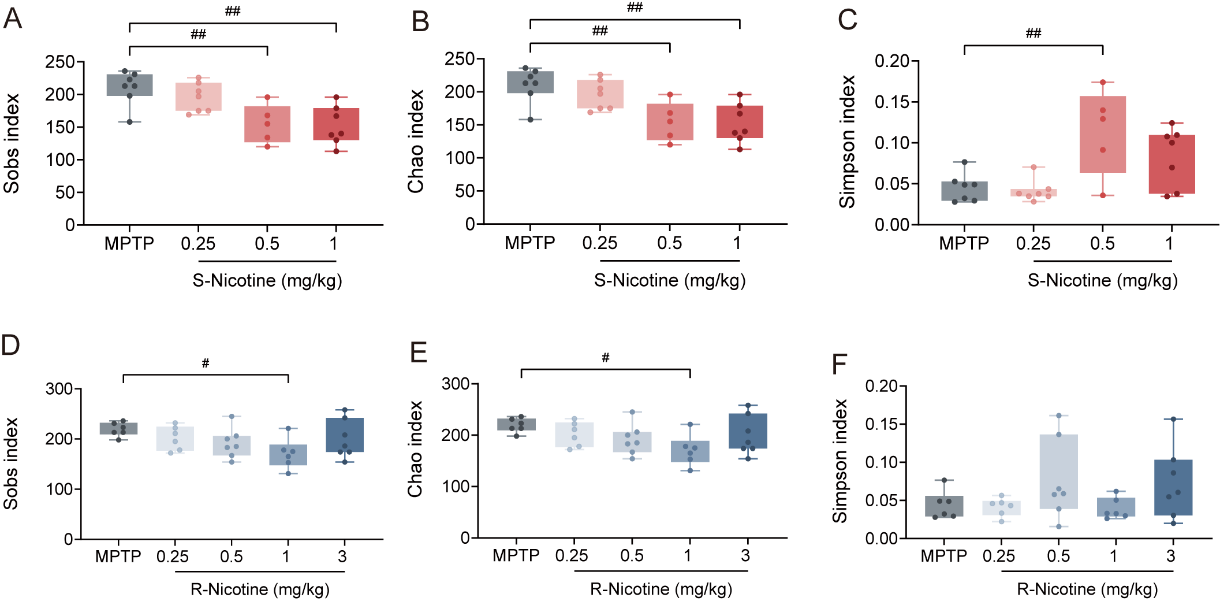


**Supplementary Figure 4.** S- and R-Nicotine Differentially Impact the Alpha Diversity of Gut Microbiota in MPTP-Treated Mice. (A-C) Gut microbiota alpha-diversity analysis in the S-Nicotine group. (A) Sobs index, (B) Chao index and (C) Simpson index. (D-F) Gut microbiota alpha-diversity analysis in the R-Nicotine group. (D) Sobs index, (E) Chao index and (F) Simpson index. Data are presented as mean ± SEM; n = 6-7 fecal samples per group. One-way ANOVA followed by Dunnett’s *post hoc* test: # *p* < 0.05, ## *p* < 0.01, ### *p* < 0.001 vs. MPTP group.


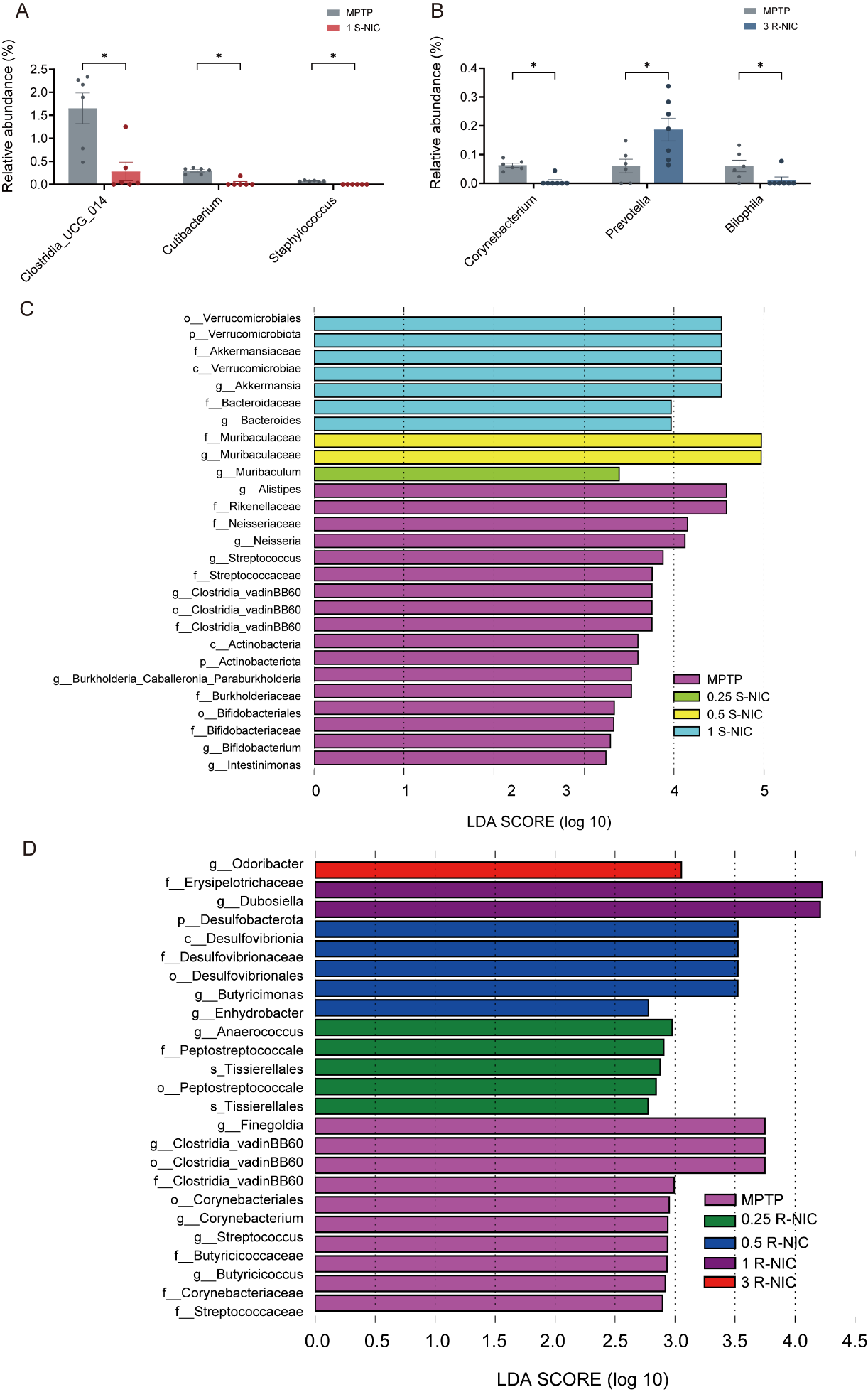


**Supplementary Figure 5.** S- and R-Nicotine Differentially Impact the Composition and Relative Abundance of Key Taxa in the Gut Microbiota. (A) Relative abundance of key bacterial genera in the S-Nicotine group. Comparisons of Clostridia_UCG_014, *Cutibacterium*, and *Staphylococcus* levels between the MPTP and 1 mg/kg S-Nicotine treated groups. (B) Relative abundance of key bacterial genera in the R-Nicotine group. Comparisons of *Corynebacterium*, *Prevotella*, and *Bilophila* levels between the MPTP and 3 mg/kg R-Nicotine treated groups. (C–D) Linear discriminant analysis (LDA) effect size (LEfSe) analysis. Identification of typical microbial taxa in the (C) S-Nicotine and (D) R-Nicotine experimental groups. Data are presented as mean ± SEM. For 16S rRNA sequencing, n = 6-7 fecal samples per group. The Kruskal-Wallis test was employed to identify significant differences, with an LDA score threshold of > 2.50. Unpaired student’s *t*-tests: **p* < 0.05, ***p* < 0.01, ****p* < 0.001 vs. MPTP group.


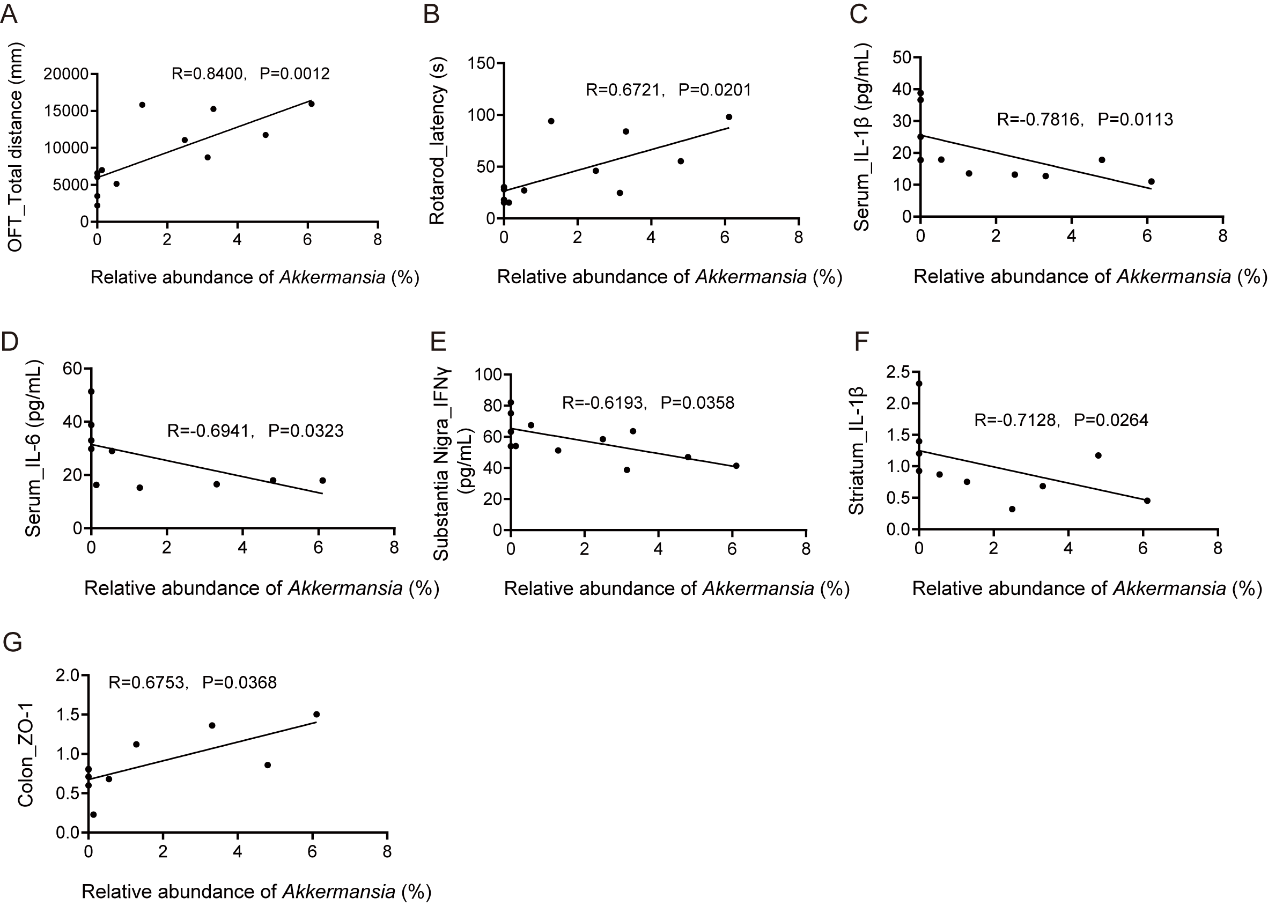


**Supplementary Figure 6.** Correlation Analysis Between *Akkermansia* Abundance and Parkinson's Disease-Related Pathological Indices. (A-G) Spearman correlation between *Akkermansia* abundance and the PD-related parameters. Relevant parameters include OFT total distance (A), rotarod latency (B), serum IL-1β (C) and IL-6 (D), substantia Nigra IFN-γ (E), striatum IL-1β (F), and colon ZO-1(G).
