# Supplementary material for: Stereoselective effects of nicotine enantiomers on the gut-brain axis and neuroinflammation in a mouse model of Parkinson’s disease
Source: Front Aging Neurosci. 2026 May 13;18:1823372. doi: 10.3389/fnagi.2026.1823372 (PMC13212218; doi:10.3389/fnagi.2026.1823372)
Supplement: Supplementary file 2 [file Data_Sheet_2.DOCX]

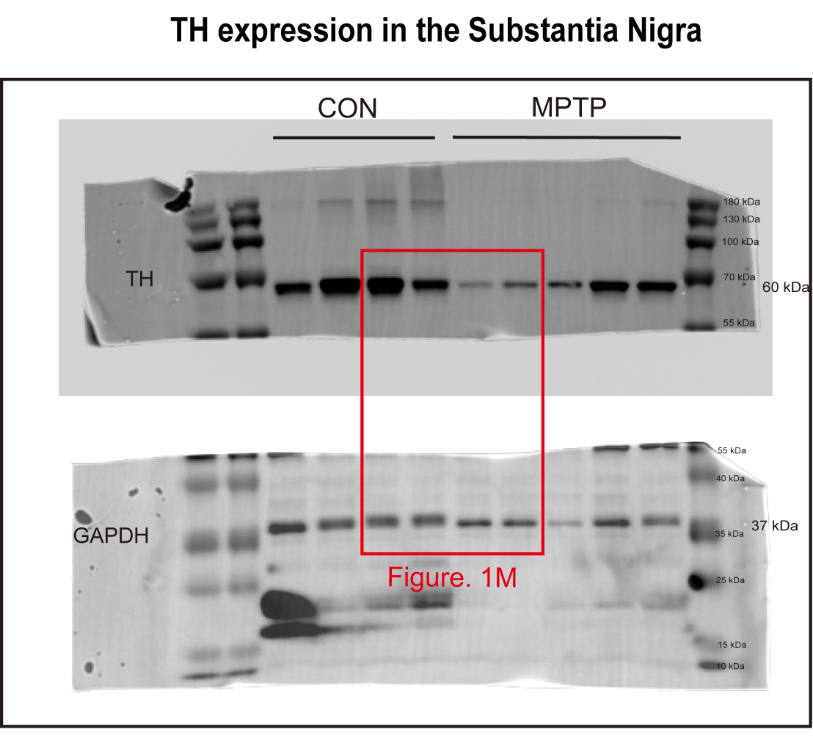


**Full uncropped Blots image(s) for TH and GAPDH in Figure.1M.**

The red box indicates the example diagram used in Figure. 1M.


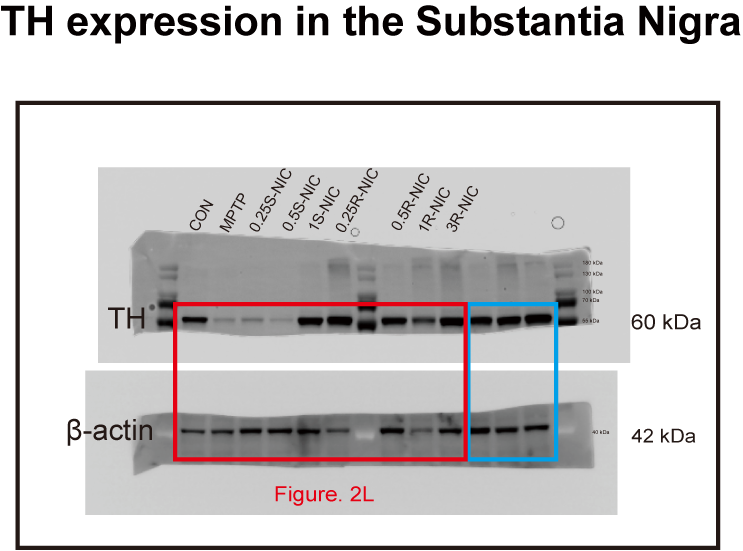


**Full uncropped Blots image(s) for TH and GAPDH in Figure.2L.**

The red box indicates the example diagram used in Figure. 2L. The blue box indicates medications not reported in this study.


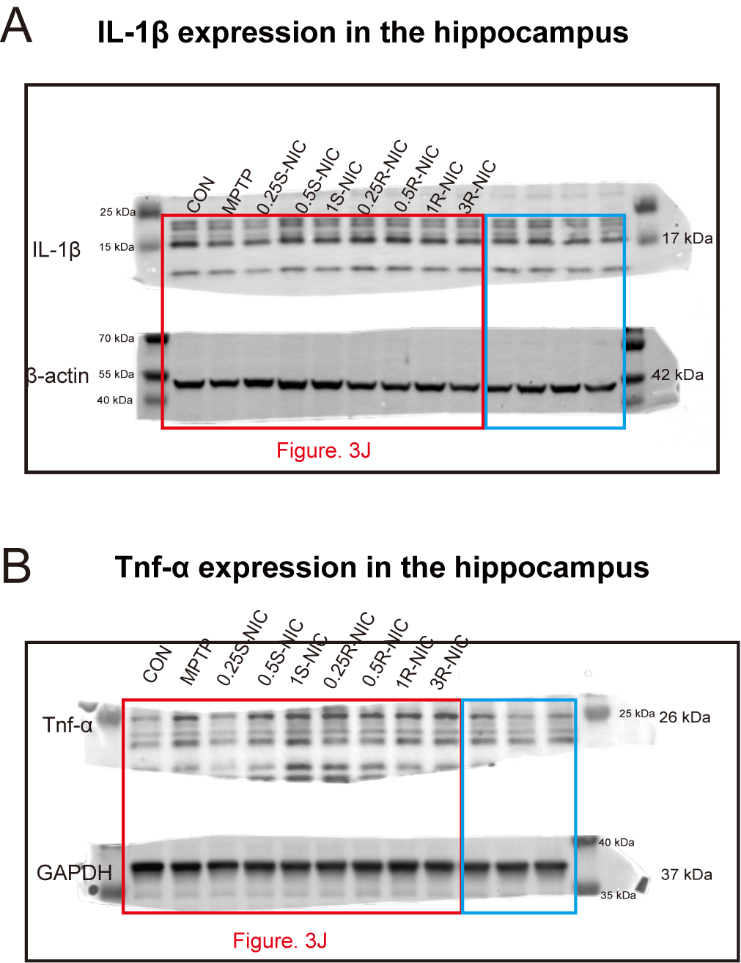


**Full uncropped Blots image(s) for IL-1β, TNF-α and GAPDH in Figure.2L.**

The red box indicates the example diagram used in Figure. 3J. The blue box indicates medications not reported in this study.


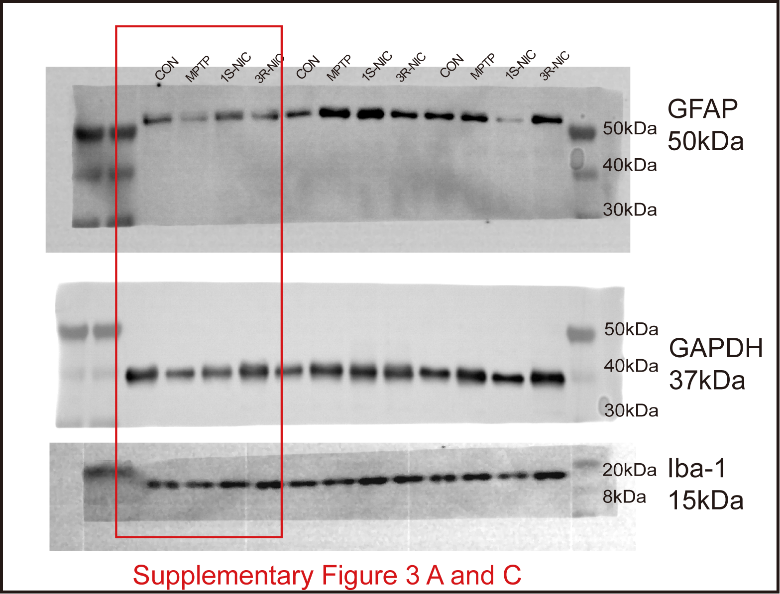


**Full uncropped Blots image(s) for GFAP, Iba1 and GAPDH in Supplementary Figure 3A and C.**

The red box indicates the example diagram used in Supplementary Figure 3A and C. The blue box indicates medications not reported in this study.
